# Self-assembling macrocyclic pillar[5]arene into toroidal, Möbius-strip-like nanoring and circularly polarized luminescence

**DOI:** 10.1093/nsr/nwaf280

**Published:** 2025-07-10

**Authors:** Jie Lu, Yuan Wang, Jingjun Jin, Minghua Liu

**Affiliations:** Key Laboratory of Colloid, Interface and Chemical Thermodynamics Institute of Chemistry, Chinese Academy of Sciences, Beijing 100190, China; Key Laboratory of Colloid, Interface and Chemical Thermodynamics Institute of Chemistry, Chinese Academy of Sciences, Beijing 100190, China; Key Laboratory of Colloid, Interface and Chemical Thermodynamics Institute of Chemistry, Chinese Academy of Sciences, Beijing 100190, China; Key Laboratory of Colloid, Interface and Chemical Thermodynamics Institute of Chemistry, Chinese Academy of Sciences, Beijing 100190, China

**Keywords:** pillar[5]arene, planar chirality, toroid, Möbius strip, circularly polarized luminescence

## Abstract

The unique spatial structure of macrocyclic molecules presents significant challenges in achieving precise chirality control across multiple hierarchical assembly levels, particularly in the fabrication of topologically chiral nanostructures. In this study, we functionalized pillar[5]arene with amphiphilic glutamide units and explored their self-assembly behavior, successfully obtaining chiral nanotoroids and Möbius-strip-like nanostructures. Through a combination of spectroscopic analysis, electron microscopy, scanning electron microscopy and atomic force microscopy, we elucidated the underlying mechanism driving molecular aggregation to the formation of toroidal topologies with well-defined chirality. These nanostructures exhibit blue circularly polarized luminescence (CPL). Notably, when achiral luminophores such as TPPS or CBS are adsorbed onto the toroidal nanostructures, they are induced to emit red or blue CPL, respectively, while the other vesicular or spherical assemblies could not. This work bridges the gap between molecule-level macrocycle chirality and nanoscale topological chirality, offering new possibilities for the design of advanced chiroptical materials.

## Teaser

This study demonstrates a novel strategy to guide supramolecular macrocycles into nanoscale chiral topological toroids, establishing hierarchical self-assembly pathways for advanced chiroptical materials

## INTRODUCTION

Macrocyclic molecules, including crown ethers, cyclodextrins, calixarenes, cucurbiturils and pillararenes, represent the cornerstone of supramolecular chemistry [1–5]. Their cyclic architectures and molecular recognition capabilities make them ideal building blocks for constructing functional nanomaterials [6–9]. Furthermore, mechanically interlocked systems such as catenanes, rotaxanes, and trefoil knots expand the structural diversity of macrocycles, introducing unique topological features and dynamic behaviors [10–12]. However, the inherent rigidity and spatial constraints of macrocyclic molecules pose significant challenges in their hierarchical self-assembly into higher-order nanostructures. In particular, the controlled formation of chiral topological nanorings from macrocycles remains largely unexplored.

Among these macrocycles, pillar[n]arenes composed of rigid, conjugated hydroquinone (or hydroquinone ether) units linked by para-bridging methylene groups have emerged as a prominent class of supramolecular hosts since their discovery by Ogoshi *et al.* [13–15]. Their symmetric yet tunable structure not only facilitates host–guest interactions, but also confers planar chirality due to the restricted rotation of benzene rings. In 2010, Ogoshi's group first demonstrated the planar chirality of pillar[5]arenes and exploited this property to design multi-stimuli-responsive chiral switches [16–18]. Since then, numerous studies have explored the chiroptical properties and applications of pillar[5]arenes [19,20]. Beyond their role as host molecules, pillararenes have also garnered attention as supramolecular building blocks for self-assembly. A diverse array of nanostructures, including helical nanowires, nanotubes and fibers, have been successfully constructed [21–23]. Nevertheless, the formation of well-defined nanoring topologies remains rare, presenting both a fundamental challenge and an exciting opportunity in the field of supramolecular nanotechnology.

As an important class of chiral assemblies, cyclic topological nanostructures such as toroids and Möbius strips have attracted significant attention in supramolecular chemistry [24,25]. The construction of such topological nanorings relies critically on inducing curvature in assembled nanofibers, in which chiral induction effects can play a key role [26,27]. In our previous work, we successfully fabricated nanoscale toroids [28] and Möbius strips [29] by using functional π-conjugated systems coupled with chiral amphiphiles. However, the assembly of macrocyclic molecules into well-defined chiral toroidal or Möbius-strip topologies remains a substantial challenge.

**Scheme 1.**
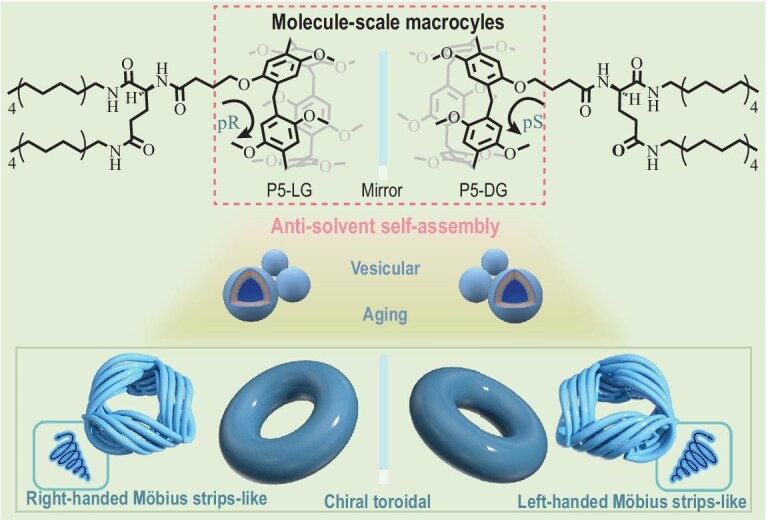
Schematic diagram of **P5-LG/DG** self-assembly into nanoscale toroidal and Möbius-strip-like nanoarchitecture.

In this study, we designed chiral amphiphilic pillar[5]arene derivatives (**P5-DG/LG**) by conjugating glutamide units to the macrocyclic framework and systematically investigated their self-assembly behavior and chiroptical properties. Remarkably, in tetrahydrofuran (THF)/water mixed solvents, these compounds spontaneously organized into chiral toroidal nanostructures and Möbius-strip-like nanorings (Scheme 1) through synergistic non-covalent interactions, including hydrogen bonding, van der Waals forces and π–π stacking. The assembly process exhibited intriguing solvent-dependent evolution: initially, molecules aggregated under solvophobic effects to form stable bilayer vesicles; upon an increase in the water content to 70%, these vesicles underwent structural reorganization into toroidal topologies, with some adopting distinct Möbius-strip-like conformation through the dynamic reconfiguration of intermolecular interactions.

Notably, the resulting nanostructures displayed structure-dependent luminescent properties: only the toroidal assemblies exhibited strong blue circularly polarized luminescence (CPL), while other aggregate forms showed no CPL activity. Furthermore, these chiral toroids could serve as supramolecular templates, inducing achiral fluorescent dyes (CBS and TPPS) to emit blue and red CPL, respectively—a phenomenon unattainable with conventional assemblies. This work establishes a novel strategy for constructing hierarchical chiral topologies from macrocyclic building blocks, spanning from molecular to nanoscale. In addition, it demonstrates precise control over chiroptical properties through solvent-mediated structural regulation, opening up new possibilities for designing functional chiral nanomaterials.

## RESULTS AND DISCUSSION

### Synthesis and characterization of molecules

For the synthesis of the chiral amphiphilic **P5-DG/LG**, the pillararene derivative **P5-COOEt** was initially prepared according to a previously established method [15]. Following hydrolysis, a glutamine derivative modified with a long alkyl chain was introduced via an amide condensation reaction. The structural integrity of the synthesized molecules was rigorously characterized by using ^1^H nuclear magnetic resonance (NMR), ^13^C NMR and high-resolution mass spectrometry (Figs S1–S12). Comprehensive analysis of the experimental data unequivocally confirmed that the molecular architecture of **P5-DG/LG** aligns precisely with the structure illustrated in Scheme 1.

### Self-assembly of macrocyclic molecules into nanoscale toroid and Möbius-strip-like nanostructure

As effective gelaton agents, many derivatives of LG/DG exhibit excellent self-assembly properties [30,31]. **P5-DG** showed a solvent-dependent self-assembly properties. When heated to dissolve in various organic solvents (1.0 mg/500 mL), including cyclohexane, CCl_4_, 1,2-dichloroethane, dichloromethane, tetrahydrofuran, chloroform, acetone, ethanol, acetonitrile, methanol and dimethylsulfoxide (DMSO), and then allowed to cool naturally to room temperature, **P5-DG** formed a gel only in acetonitrile and DMSO, while it remained as a dispersion or solution in other solvents. Among these solvents, THF can completely dissolve **P5-DG** even at room temperature. However, when water was added into the THF solution, self-assembly was triggered and interesting nanostructures were observed. Thus, we focused on the self-assembly of **P5-DG** in the mixed solvents of THF/water by using the anti-solvent assembly process at room temperature.

**Figure 1.**
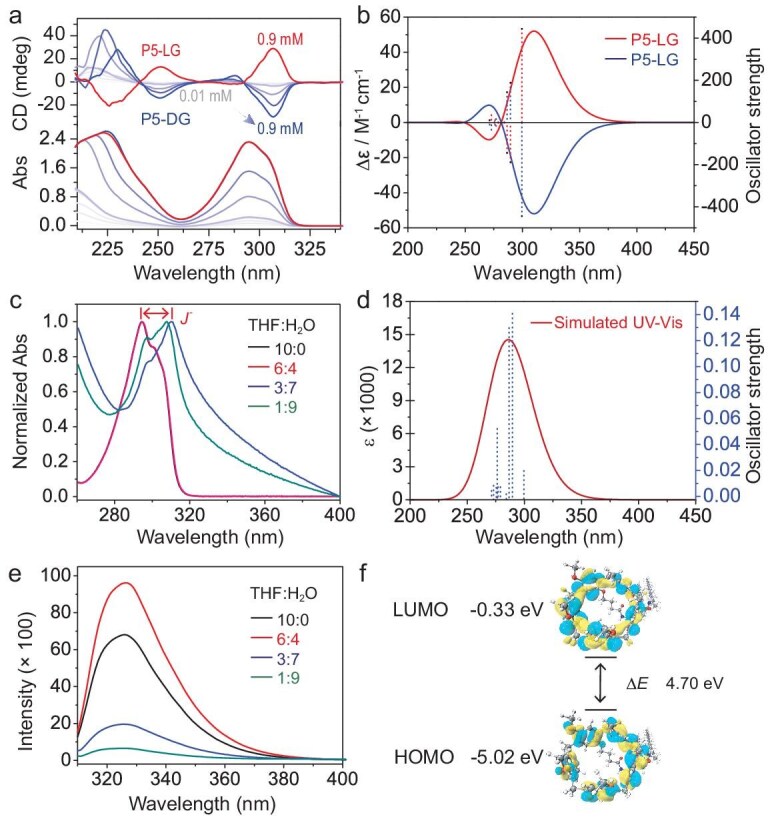
(a) CD spectra (top) and UV–vis absorption (Abs) (bottom) of **P5-LG** and different concentrations (0.01, 0.03, 0.06, 0.09, 0.1, 0.3, 0.6 and 0.9 mM, from top to bottom) of **P5-DG** in THF and the cuvette path length is 1 mm. (b) Theoretical CD spectra of **P5-DG/LG**; the calculated oscillator strengths and rotatory strengths are also indicated by dotted vertical lines. (c) Abs spectra of **P5-DG** in different solvents. (d) Simulated UV–vis absorption (Abs) of **P5-DG**. (e) Emission (excited at 295 nm, Ex bandwidth: 5 nm; Em bandwidth: 5 nm) spectra of **P5-DG** in different solvents. (f) Lowest unoccupied molecular orbital (LUMO) and highest occupied molecular orbital (HOMO) of **P5-DG**.

To determine the optimal assembly concentration, concentration-dependent UV–vis and CD experiments (Fig. 1a and Fig. S13a) were conducted on THF solutions of **P5-DG** and **P5-LG**, respectively. The UV–vis spectra (Fig. 1a) showed absorption bands at ∼295 nm, corresponding to the π–π* transition of benzene. This result was consistent with the simulated UV–vis absorption data (Fig. 1d and Fig. S14) from theoretical calculations for **P5-DG/LG**. As the concentration increased, a negative Cotton effect emerged at 307 nm and the intensity of the CD signal gradually increased. This effect is attributed to the gradual assembly of **P5-DG**, in which the point chirality of the glutamic acid unit induces planar chirality in pillar[5]arene [32]. Furthermore, the excellent agreement between the theoretically simulated and experimentally measured CD spectra of **P5-DG** (Fig. 1a and b, and Fig. S13b) provides strong support for this conclusion. For further characterization of the aggregation process, a concentration of 0.3 mM was selected for subsequent experiments.

The CD spectra of **P5-DG** and **P5-LG** exhibited mirror-image symmetry (Fig. S13b), indicating that enantiopure **P5-DG** and **P5-LG** with planar chirality had been obtained in THF. Subsequently, varying amounts of ultrapure water were added to the well-dissolved molecules and the UV–vis spectral data are shown in Fig. 1c and Figs S13c and S15a and b. When the water content ratio was <50%, the UV–vis absorption spectrum showed little change. However, when the water content increased to 70%, a significant red shift appeared, indicating that the pillar[5]arene units were tending toward *J*-type aggregation in the water-rich solution [15,33]. The compounds are luminescent due to the pillar[5]arene units (Fig. 1f). Fluorescence spectra (Fig. 1e and Figs S13d and S15c and d) showed that, as the water content gradually increased, the fluorescence emission intensity at 330 nm first increased and then decreased. At 40% water content, the fluorescence intensity reached its maximum, which can be attributed to the aggregation-induced emission (AIE) behavior of the pillar[5]arenes [33]. Combining the UV–vis and fluorescence spectra, we selected H_2_O/THF mixtures with water contents (v/v) of 40%, 70% and 90% for more detailed self-assembly studies.

After adding varying volumes of ultrapure water to the THF solution of **P5-DG**, a transparent homogeneous phase formed at 40% water content (Fig. S16a), while turbid dispersions were obtained at 70% and 90%, respectively. After standing for 24 hours, the state of the 40% water content solution remained unchanged (Fig. S16b). The 70% water dispersion exhibited some precipitation but remained turbid, while the 90% dispersion showed significant precipitation and became clear in the upper layer. After 24 hours of further standing, the 70% water content emulsion clarified and precipitated a large amount of solid (Fig. S16c). An obvious Tyndall effect (Fig. S17a) and dynamic light scattering (DLS) data (Fig. S17b) at 293 K also clearly supported the formation of particles with sizes of approximately 400, 800 and 1720 nm, respectively.

**Figure 2.**
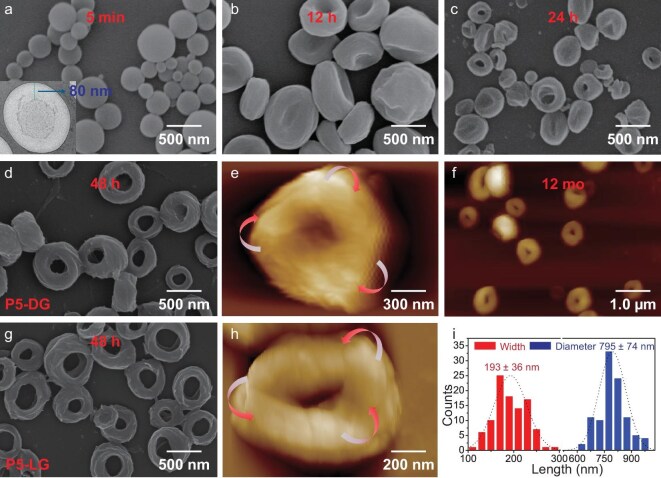
SEM images of **P5-DG** in THF/H_2_O (v/v = 3/7) after (a) 5 minutes (the lower left corner shows a transmission electron microscopy (TEM) image of the negatively stained sample), (b) 12 hours, (c) 24 hours and (d) 48 hours. (e) AFM image of **P5-DG** in THF/H_2_O (v/v = 3/7) after 48 hours. (f) AFM image of **P5-DG** following 12 months of aging in THF/H_2_O (v/v = 3/7). (g) SEM images of **P5-LG** in THF/H_2_O (v/v = 3/7) after aging for 48 hours. (h) AFM images of **P5-LG** in THF/H_2_O (v/v = 3/7) after 48 hours. (i) Statistical analysis of the toroidal width and diameter of **P5-DG**.

After centrifugation, the same volume of solvent was added to the precipitate and scanning electron microscopy (SEM) experiments were subsequently performed. The experimental data showed that **P5-DG** assembled into a vesicular structure with a wall thickness of ∼5.5 nm (Figs S18a and S19a and c) at 40% water content. Interestingly, a toroidal topology (Fig. S18b) formed when the water content reached 70% and larger spherical aggregates (Fig. S18c) were observed at 90% water content. The formation process of the toroidal topology structure was investigated in detail by using time-dependent SEM experiments. As shown in Fig. 2, when water was added to the THF solution of **P5-DG**, the aggregates exhibited as a vesicular structure within 5 min. The vesicle has a wall thickness of ∼80 nm (Fig. 2a and Fig. S19b and d). As the assembly time increased to 12 hours, the vesicular aggregates began to concave at the center, forming an erythrocyte-like structure (Fig. 2b). After 24 hours, some assemblies ruptured in the middle to form a toroidal topology (Fig. 2c). After 48 hours, the aggregates exhibited a complete toroidal topological structure (Fig. 2d and Fig. S18b). Long-term stability assessment via atomic force microscopy (AFM) (Fig. 2f and Fig. S20b and c) and SEM (Fig. S20a) after 12 months of aging confirmed the structural integrity of the toroidal architecture, with no observable degradation or morphological alterations. Morphological statistics analysis revealed an average outer diameter of 795 ± 74 nm and a toroidal width of 193 ± 36 nm (Fig. 2i). Through AFM measurements, we even observed some left-handed Möbius-strip-like structures formed by **P5-DG** (Fig. 2e). The enantiomeric **P5-LG** of **P5-DG** also assembled into a toroidal topology (Fig. 2g and Fig. S18e) at a water content of 70%, with a morphological statistical analysis showing an average outer diameter of 803 ± 169 nm and a toroidal width of 235 ± 53 nm (Fig. S21). In addition, AFM revealed that the enantiomeric **P5-LG** self-assembled into a right-handed Möbius-strip-like structure (Fig. 2h).

**Figure 3.**
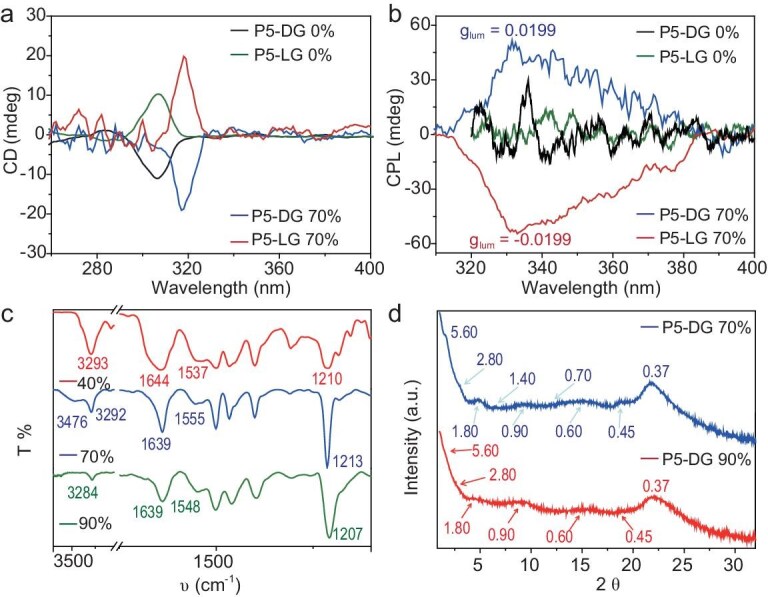
(a) CD spectra and (b) CPL (Ex = 285 nm) spectra of **P5-DG** and **P5-LG** in different solvents. (c) Partial FT–IR spectra of **P5-DG** from different volume ratios of THF/H_2_O solutions. (d) XRD patterns of **P5-DG** assemblies in THF/H_2_O (v/v = 1/9) and THF/H_2_O (v/v = 3/7).

Due to the ability of point chirality to induce planar chirality in pillar[5]arenes [34], CD and CPL experiments were performed for **P5-DG** and **P5-LG** assemblies. The CD data (Fig. 3a and Fig. S22a) revealed that the spectral signals of the four samples, at the same concentration, were clearly different. In pure THF solution, **P5-DG** showed a relatively strong negative Cotton effect at ∼306 nm, while relatively weak negative Cotton signals appeared at around 307 and 314 nm at water contents of 40% and 90%, respectively. In contrast, a strong negative Cotton effect appeared at ∼318 nm when the water content was 70%. Strong CPL signals (Fig. 3b and Fig. S22b) were detected at 331 nm only when the water content reached 70%, with a |g_lum_| value as high as 0.0199 (Fig. 3b and Fig. S23a), which might be the highest value for the parent pillararenes [4,5,14,18,19,32,35–50] (Fig. S23b and Table S1). When the water content was 40% and 90%, respectively, only very weak CPL signals were detectable. Thus, the strong |g_lum_| observed may be attributed to the toroidal topological structure. Moreover, the CD data (Fig. 3a) and CPL data (Fig. 3b) of **P5-LG** showed mirrored spectra, supporting the handedness of the assembled chiral nanostructures.

### Mechanism for the formation of the toroid nanostructures

In order to understand why the toroid nanostructures were formed, we further investigated the Fourier transform infrared (FT–IR), X-ray diffraction (XRD), time-dependent DLS and 1D-selective gradient nuclear Overhauser effect spectroscopy (NOESY) to monitor the change in molecular interaction and packings. FT–IR spectra of three **P5-DG** assemblies were measured (Fig. 3c and Fig. S24c). The toroidal structure formed by **P5-DG** exhibited clear vibration bands of N–H at 3476 and 3292 cm^−1^, while only one N–H vibration band appeared at 3293 or 3284 cm^−1^ for vesicular or spherical aggregates, respectively. The N–H vibration at 3476 cm^−1^ could be ascribed to the N–H stretching of free amide moiety (Fig. S25), whereas the band at 3284 (or 3292 and 3293) cm^−1^ could be ascribed to the hydrogen-bonded N–H of the other two amide groups. This indicated that hydrogen bonds are quite different in toroid from those in vesicles and nanospheres. In vesicles and nanospheres, the amide groups are wholly H-bonded, while, in toroid, some free amide existed. The aggregates obtained from the enantiomer **P5-LG** in solutions with different water contents (Fig. S24a and d) were basically similar to the data of **P5-DG**.

**Figure 4.**
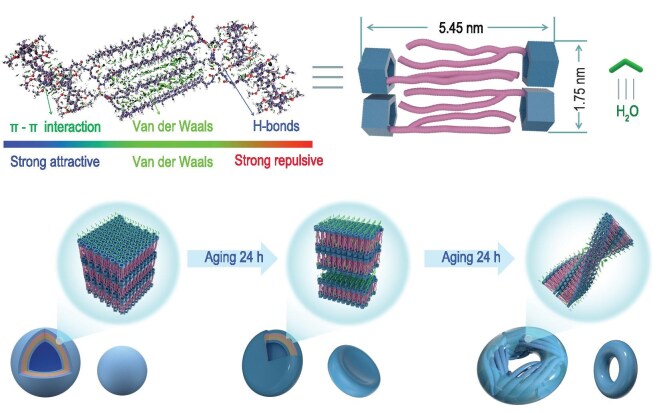
Simulation results of **P5-DG** assembly conformation and cartoon schematic illustration of its topologically chiral cyclization in THF/H_2_O (v/v = 3/7) solution.

The XRD of the assembled structures of **P5-DG** and **P5-LG** were further investigated (Fig. 3d and Fig. S24b). As the data for **P5-DG** and **P5-LG** are essentially similar, the XRD data of **P5-DG** will be used as an example for analysis. The toroid topology formed by **P5-DG** is reflected in the XRD profiles, which show *d*-spacings of 5.60, 2.80, 1.80, 1.40, 0.90, 0.70, 0.60 and 0.45 nm. For *d*-spacings of 5.60, 2.80, 1.40, 0.90 and 0.70 nm, we observe that the ratios 1, 1/2, 1/4, 1/6 and 1/8 correspond to the lamellae structures with a spacing of 5.60 nm. Other ratios of 1, 1/2, 1/3 and 1/4 for *d*-spacings of 1.80, 0.90, 0.60 and 0.45 nm are also observed, which are also layered structures with spacing of 1.8 nm. As the length of 5.6 nm exceeds that of a single molecule (3.86 nm) but is less than two molecular lengths, a bilayer structure with interdigitated alkyl chains is proposed (Fig. 4 and Fig. S26a). The other layer spacing of 1.8 nm is attributed to the layered structure formed by the parallel arrangement of the rigid pillar[5]arene rings (Fig. 4). The similarly layered structure observed in the toroidal suggests that the bilayer structure of **P5-DG** molecules acts as the basic unit for all the assemblies. To further understand such packing, we optimized the structures of **P5-DG** tetramers and obtained relatively stable conformations (Fig. 4 and Fig. S26). The equilibrium configuration reveals that a hydrogen bond of 1.88 Å is formed between the C=O group on amide part 3 and the N–H bond on amide part 1 (Fig. S25) and a hydrogen bond of 1.97 Å is formed between the N–H bond on amide part 3 and the C=O group on amide part 2, which is consistent with the infrared spectroscopy data. The alkyl chains of **P5-DG** molecules are stacked in parallel under the influence of van der Waals forces, forming a layered structure with a spacing of 5.45 nm (Fig. 4). The pillar[5]arene units are stacked with each other through π–π stacking, forming a layered structure with a spacing of 1.75 nm, which agrees with the XRD data well.

DLS were measured at different time intervals to monitor the change in the assemblies (Fig. S17c and d). The data revealed the progressive growth of aggregate during this process, reaching a plateau with no further size variation after 48 hours. Aligned with the SEM observations, it is suggested that, upon adding water to the THF solution of **P5-DG**, small vesicles were immediately generated via hydrophobic/hydrophilic interactions. Subsequently, driven by the hydrogen bonding, π–π stacking and hydrophobic/hydrophilic interactions, these small vesicles may gradually coalesce into larger aggregates, ultimately evolving into toroidal topological structures over time, which was confirmed by the SEM. The sizes from the SEM observation and the DLS were the same.

Furthermore, to investigate whether host–guest interactions exist between the pillar[5]arene macrocycle and the alkyl chains, we performed 1D-selective gradient NOESY experiments by selectively exciting the aromatic protons **H_a–__d_** of the pillar[5]arene unit. As illustrated in Fig. S27, strong nuclear Overhauser effect (NOE) signals were only observed between protons **H_a–d_** and other protons **H_e–l_** on the pillar[5]arene unit, while no NOE correlations were detected between these aromatic protons and the alkyl chain protons **H_m_**. These results clearly indicate that no host–guest complex is formed between the pillar[5]arene moiety and the long alkyl chains in THF/H_2_O solution.

Combining all these data together with the change in the SEM, it can be suggested that amphiphilic **P5-DG** formed the bilayer as the basic unit through the synergistic interactions of hydrogen bonding, π–π stacking and van der Waals forces. When the water content increased, the circular vesicles collapsed into toroid with the stacking of multi-bilayers (Fig. 4). During such a process, the H bonds between some amphiphiles were released in order to form the inner layer of the toroid. An important factor for forming such toroid structures may be due to the balance between the large head macrocyclic ring and the two hydrophobic alkyl chains. Such a structure is stable in a THF/water mixture and can be separated from the assemblies. Interestingly, such a unique toroid can further serve as the template for the chirality induction of achiral molecules, as discussed below.

### Chiral induction and transfer from chiral toroid to fluorescent dyes

**Figure 5.**
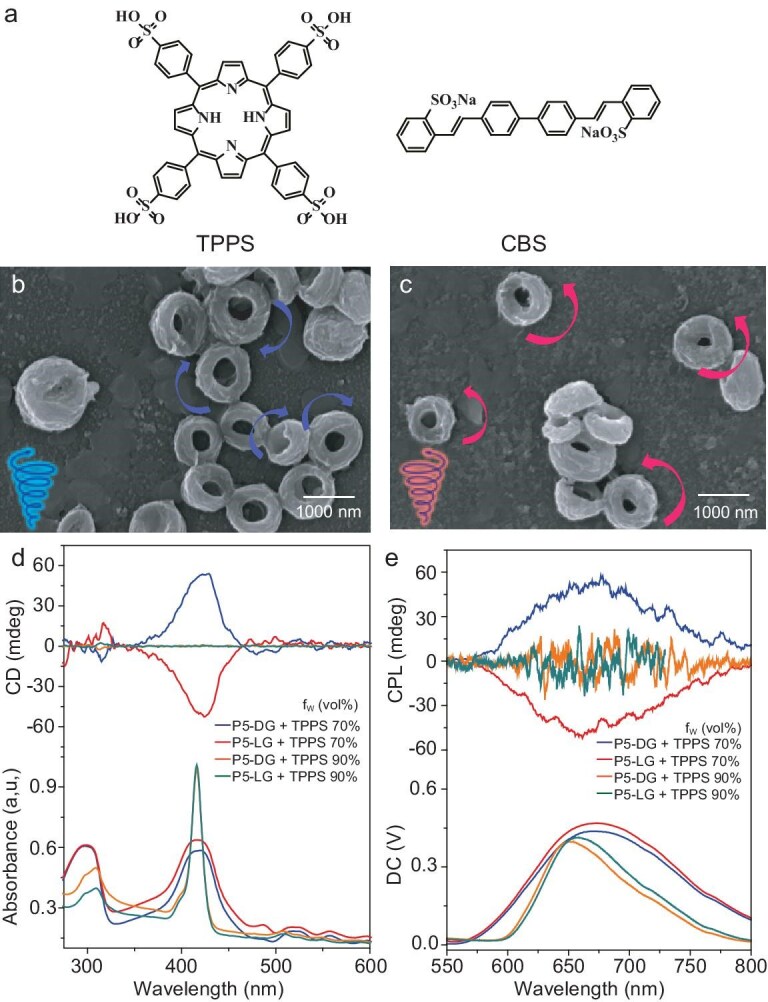
(a) Chemical structures of fluorescent dyes TPPS and CBS. SEM images of topological toroidal of (b) **P5-DG** after adsorption of TPPS and (c) **P5-LG** after adsorption of TPPS. (d) CD spectra (top) and UV–vis absorption (Abs) (bottom) and (e) CPL (Ex = 420 nm) spectra (top) and DC value (bottom) of various topological structures of **P5-DG/LG** after adsorption of TPPS.

The assembly structure plays a significant role in determining its function [15,23,30,31] and we aimed to incorporate two fluorescent molecules (TPPS and CBS, Fig. 5a) into the chiral aggregates mentioned above. The fluorescent molecules TPPS and CBS were added to the **P5-DG** or **P5-LG** systems via simple adsorption. SEM data indicated that the toroidal aggregates of **P5-DG** and **P5-LG** did not change in their topological structures after the adsorption of TPPS (Fig. 5b and c, and Fig. S28a and b) or CBS (Fig. S28c and d). Subsequently, the ground-state chirality was studied by using CD spectroscopy to verify that the chiral aggregate acted as a matrix to transfer chirality to the achiral fluorescent molecules. Figure 5d and Figs S29a and S30a show the UV–vis and CD spectra of various **P5-DG** and **P5-LG** aggregates with adsorbed TPPS or CBS. As shown in Fig. 5d and Fig. S29a, when TPPS is adsorbed by the assembly, a mirror-imaged CD signal is observed at 417 nm when the toroid structure was used as template. No CD signal is detected near 417 nm when the structure consists of spherical micelles or vesicles were used. At ∼360 nm, the CD spectrum after the adsorption of CBS by **P5-DG** shows a positive CD signal, while **P5-LG** shows a negative CD signal after CBS adsorption, consistent with the absorption band of CBS (Fig. S30a). This indicates that chirality is transferred from the toroid structure to the achiral fluorescent dye molecules. Finally, to further verify the transfer of chirality from the chiral aggregates to the fluorescent dyes, excited-state chirality was investigated by using CPL spectroscopy. Figure 5e and Figs S29b and S30b show the CPL spectra for various aggregates of **P5-DG** and **P5-LG** adsorbed with TPPS or CBS. Figure 5e shows that positive and negative CPL signals are observed at ∼670 nm after the adsorption of TPPS by **P5-DG** and **P5-LG** toroid, with corresponding g_lum_ values of ±0.0095 (Fig. S31a), respectively. Similarly, **P5-DG** exhibits a positive CPL signal near 425 nm after CBS adsorption (Fig. S30b), while **P5-LG** shows a negative CPL signal after CBS adsorption. In addition, we could only detect very weak CPL signals when a nanosphere served as the template, as shown in Fig. 5e and Fig. S29a. Therefore, it is clear that the CPL of achiral dyes is generated by adsorption onto the toroidal structures.

## CONCLUSION

In this study, we successfully synthesized **P5-DG** and **P5-LG** by connecting pillar[5]arene units with DG/LG moieties via amide bonds, enabling the transfer of point chirality from the glutamic acid units to planar chirality in the pillar[5]arene system. Furthermore, we developed an anti-solvent chiral assembly strategy to construct nanoscale toroidal and Möbius-strip-like nanostructures. When **P5-DG** or **P5-LG** was dissolved in THF followed by the addition of water, the molecules rapidly self-assembled into vesicular structures through synergistic π–π stacking, van der Waals interactions and hydrogen bonding. Over time, these vesicles evolved into toroidal topologies and even Möbius-strip-like architectures, driven by the dynamic equilibration of intermolecular forces. Notably, the chirality of the resulting toroidal structures was dictated by the stereocenters of the precursor molecules, effectively translating point chirality into planar chirality and ultimately geometric chirality. Additionally, these toroidal nanostructures served as templates for adsorbing achiral luminescent dyes. The dyes adsorbed onto the toroids exhibited CPL, whereas those on vesicular or spherical aggregates did not, underscoring the critical role of the toroidal topology in inducing chiroptical activity. This work not only demonstrates the controlled formation of chiral toroidal nanostructures, but also highlights their potential as novel platforms for chiroptical material design, opening up new avenues for advanced functional materials.

## METHODS

Experimental methods are available in the Supplementary data.

## Supplementary Material

nwaf280_Supplemental_File
