# Supplementary material for: Self-assembling macrocyclic pillar[5]arene into toroidal, Möbius-strip-like nanoring and circularly polarized luminescence
Source: Natl Sci Rev. 2025 Jul 10;12(9):nwaf280. doi: 10.1093/nsr/nwaf280 (PMC12445862; doi:10.1093/nsr/nwaf280)
Supplement: nwaf280_Supplemental_File [file nwaf280_supplemental_file.pdf]

Electronic Supporting Information

Self-assembling macrocyclic pillar[5]arene into toroidal, Möbius-strip-like nanoring and circularly polarized luminescence

Jie Lu†, Yuan Wang†, Jingjun Jin and Minghua Liu*

Table of Contents

1. Experimental Procedures 1
1.1 Chemicals and Reagents 1
1.2 Characterizations 1
1.3 Synthetic procedures of chiral molecules 3
1.4 Supplementary NMR and MS spectra 7
1.5 Sample Preparations 14
1.6 Theoretical simulation 14
2 Results and Discussion 15
2.1 Supplementary spectra of **P5-DG/LG** in different solvents 15
2.2 Supplementary photographs and DLS image of **P5-DG/LG** in different solvents 17
2.3 Supplementary SEM and TEM image of **P5-DG/LG** in different solvents 18
2.4 Supplementary CD and CPL spectra of **P5-DG/LG** 21
2.5 Supplementary IR spectra of **P5-DG/LG** 23
2.6 Supplementary Theoretical simulation of **P5-DG/LG** 24
2.7 Investigation of Host-Guest Interactions Between Pillar[5]arene Macrocycles and Long Alkyl Chains 26
2.8 Supplementary SEM and spectra of P5/dyes co-assemblies 26
REFERENCES 29
Author Contributions 30

1. Experimental Procedures

1.1 Chemicals and Reagents

1, 3, 5-Trioxane, N-(tert-butoxycarbonyl)-L-glutamic acid (Boc-L-Glu) and N-(tert-butoxycarbonyl)-D-glutamic acid (Boc-D-Glu) were purchased from TCI. 1-Octadecylamine was brought from Alfa Aesar. 1-Hydroxybenzotriazole (HOBt) was purchased from J&K. 1-(3-Dimethylaminopropyl)-3-ethylcarbodiimide hydrochloride (EDC), ethyl 4-bromobutyrate, 1,2-dichloroethane, boron trifluoride diethyl etherate, 1, 4-dimethoxybenzene, 4-methoxyphenol, dichloromethane (anhydrous, 99.9%, water ≤ 30 ppm) and trifluoroacetic acid were purchased from Innochem. Potassium carbonate (K_2CO_3), sodium hydroxide (NaOH), acetonitrile, dichloromethane (DCM), ethyl acetate (EA), petroleum ether (PE), ethanol and tetrahydrofuran (THF) were supplied by Beijing Chemical Regent company (China). Milli-Q water (18.2 MΩ•cm) was used in all cases. All the materials and solvents were bought from commercial suppliers without further purification.

1.2 Characterizations

NMR and mass spectra

^{1}H NMR (400 MHz) and ^{13}C NMR (101 MHz) spectra were recorded on a Bruker Avance 400 spectrometer with TMS as internal standard at 298K. 1D-selective gradient NOESY (700 MHz) spectra were recorded on a Bruker NOE 700 spectrometer at 298K. Matrix assisted laser desorption/ionization time of fight mass spectrometry (MALDI-FTICR-MS) were performed on Bruker-Autoflex III.

Ultraviolet and visible absorption spectroscopy (UV-vis)

UV-vis spectra were obtained either simultaneously with the CD spectra on the CD spectrometer or separately on Hitachi UV-2600 UV-vis absorption spectrometer. Solution samples were loaded in a quartz cuvette with 1 mm optical path. The solid samples were loaded in 0.1 mm quartz cuvette for FL spectra measurement.

Fluorescence spectra (FL)

Fluorescence spectra were measured on F-4500 fluorescence spectrophotometer. Solution samples were loaded in a quartz cuvette with 1 mm optical path. The solid

samples were loaded in 0.1 mm quartz cuvette for FL spectra measurement.

Circular dichroism (CD) spectrum

Circular dichroism (CD) spectra were obtained using JASCO J-815 spectrophotometer. Solution samples were loaded in a quartz cuvette with 1 mm optical path. The solid samples were loaded in 0.1 mm quartz cuvette for FL spectra measurement.

Circularly polarized luminescence spectrum (CPL)

Circularly polarized luminescence (CPL) spectra were obtained using JASCO CPL-300 spectrophotometer. The solid samples were loaded in 0.1 mm quartz cuvette for CPL spectra measurement. Solution samples were loaded in a quartz cuvette with 1 mm optical path. The luminescence dissymmetry factor (g_{lum}) spectra were transferred from CPL spectra using the SpectraManager software of JASCO. The glum was used to quantify the extent of chiral fluorescence dissymmetry, given by the formula $g_{lum} = 2\ (\mathrm{I}_L - I_R)/(I_L + I_R)$, where I_L and I_R represent the intensities of left and right circularly polarized light, respectively.

Scanning electron microscopy (SEM)

SEM was performed on S4800 (Hitachi, Japan) with an accelerating voltage of 10 kV and a working current of 10 μA to *ex* situ characterize the assemblies. Before SEM measurement, the samples on silicon wafers were coated with a thin layer of Pt to increase the contrast.

Transmission electron microscopy (TEM)

Transmission electron microscope (TEM) experiments were performed with a JEOL 2010Plus microscope and HT7700.

Atomic force microscope (AFM)

AFM images were recorded on a f Dimension FastScan (Bruker) with a silicon cantilever in the tapping mode (silicon cantilevers of 30 μm length with typical resonant frequencies of 400 kHz and with a spring constant of 4 $N{\bullet}m^{-1}$). All AFM images are shown in the height mode without any image processing except flatting.

Powder X-ray diffraction (XRD) measurements

X-ray diffraction (XRD) patterns were achieved on a Rigaku D/Max-2500 X-ray diffractometer (Japan) with Cu K_{α} radiation (λ = 1.54 Å), which were operated at a voltage of 45 kV and a current of 100 mA. Samples were cast on silicon substrates and dried under vacuum for XRD measurements.

Fourier Transform Infrared Spectrometer (FT-IR)

FT-IR spectra were recorded on KBr pellets using JASCO FTIR-660 spectrometer.

Dynamic Light Scattering (DLS)

A zetasizer Nano-ZS was used to analyze the size distributions of molecules in solution.

1.3 Synthetic procedures of chiral molecules

1.3.1 Synthesis of P5-COOEt.

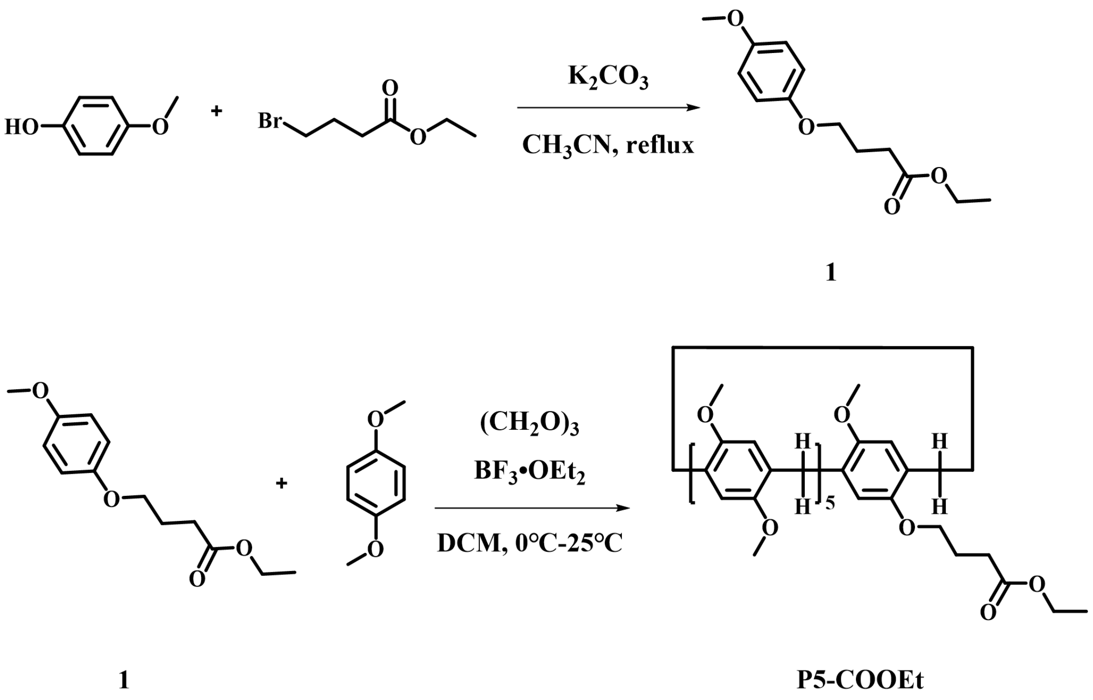

Scheme S1. Synthetic route of **P5-COOEt**.

Firstly, the precursors **1** were synthesized and characterized. Potassium carbonate (13.90 g, 0.10 mol) and 4-methoxyphenol (12.41 g, 0.10 mol) were dispersed in 150 mL acetonitrile. Next, ethyl 4-bromobutanoate (23.40 g, 0.12 mol) was added dropwise to the mixture, refluxed 24 hours under a nitrogen environment. After the organic solution was removed and water was added, extracted with dichloromethane and ethyl acetate. At last, 19.30 g pure target molecule **1** (81.14% yield) was obtained

through column chromatography.

^{1}H NMR (400 MHz, $CDCl_3$, δ, ppm): 6.82 (s, 4H), 4.14 (q, *J* = 7.2 Hz, 2H), 3.95 (t, *J* = 6.0 Hz, 2H), 3.76 (s, 3H), 2.50 (t, *J* = 7.2 Hz, 4H), 2.11 - 2.05 (m, 2H), 1.26 (t, *J* = 7.2 Hz, 3H).

^{13}C NMR (101 MHz, $CDCl_3$, ppm): 173.28, 153.86, 153.01, 115.46, 114.65, 77.40, 77.08, 76.76, 67.41, 60.40, 55.72, 30.86, 24.78, 14.23.

EI-MS: calculated for $C_{13}H_{18}O_4$, $[M]^+$ 238.1205; found $[M]^+$ 238.1195.

Precursors **1** (1.00 g, 4.20 mmol), 1, 4-dimethoxybenzene (5.81 g, 42.05 mmol) and 1, 3, 5-trioxane (1.39 g, 46.33 mmol) were dissolved in 150 mL anhydrous dichloromethane under nitrogen atmosphere. Then, boron trifluoride diethyl etherate ($BF_3{\cdot}O(C_2H_5)_2$, 2.98 g, 21.01 mmol) was added to the mixture in an ice/water bath, and the mixture was stirred at room temperature for 2 h. The solution was poured into water, extracted with dichloromethane. At last, 1.21 g pure target molecule **P5-COOEt** (33.85% yield) was obtained through column chromatography.

^{1}H NMR (400 MHz, $CDCl_3$, δ, ppm): 6.79-6.73 (m, 9H), 6.71 (s, 1H), 4.00 (q, *J* = 7.2 Hz, 2H), 3.86 (t, *J* = 6.0 Hz, 2H), 3.76-3.73 (m, 10H), 3.66-3.63 (m, 27H), 2.54 (t, *J* = 7.6 Hz, 2H), 2.12-2.06 (m, 2H), 1.07 (t, *J* =7.2 Hz, 3H).

^{13}C NMR (101 MHz, $CDCl_3$, δ, ppm): 173.32, 150.87, 150.83, 149.95, 128.36, 128.34, 128.31, 128.28, 128.21, 128.19, 114.95, 114.26, 114.22, 114.18, 114.14, 114.05, 77.41, 77.09, 76.77, 67.45, 60.43, 55.84, 55.82, 55.79, 55.77, 55.75, 31.69, 31.39, 31.23, 29.69, 25.23, 22.60, 22.42, 14.00.

MALDI-FTICR-MS: calculated for $C_{50}H_{80}O_{12}$, $[M]^+$ 850.3922; found $[M]^+$ 850.3824, $[M+K]^+$ 889.3559.

1.3.2 Synthesis of P5-LG/DG.

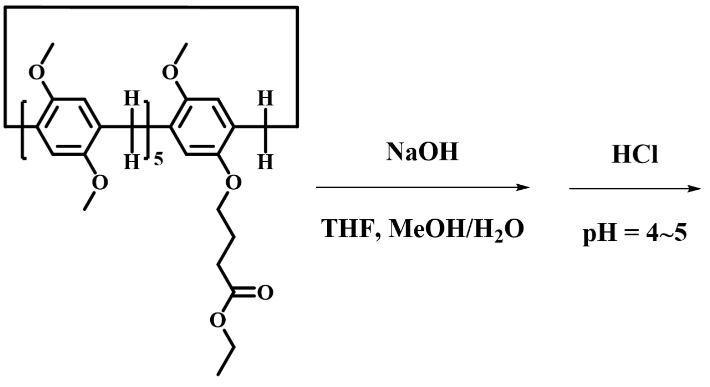

P5-COOEt **P5-COOH**

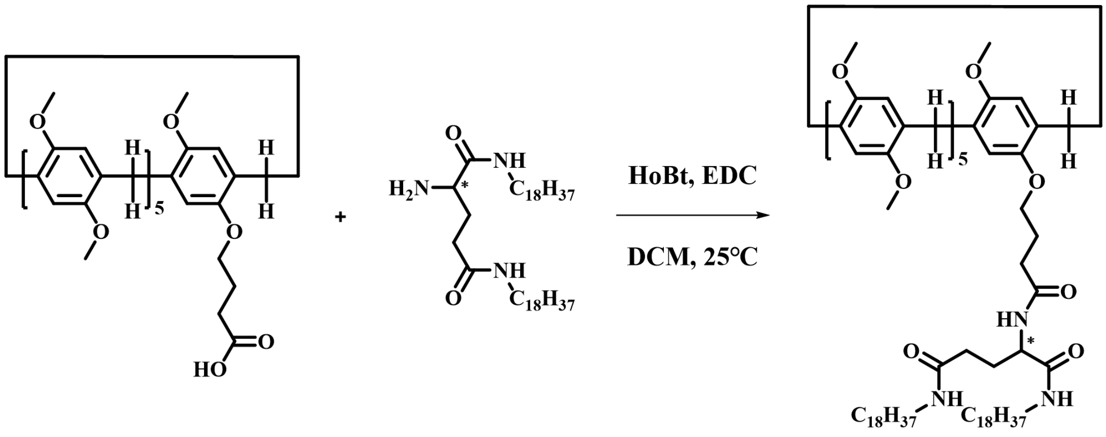

P5-COOH **LG/DG** **P5-LG/DG**

Scheme S2. Synthetic route of **P5-LG/DG**.

Firstly, the precursors **LG** and **DG** were synthesized and characterized according to the previously reported method in our group.[S1-2] **P5-COOEt** (1.20 g, 1.41 mmol) and NaOH (0.282 g, 7.05 mmol) were dissolved in 110 mL THF/MeOH/H_2O (v/v/v = 1: 5: 5). After stirring at room temperature for 12 h, the solvent was removed by vacuum distillation. Then 50 mL of distilled water was added and the pH is adjusted to 4~6 with diluted hydrochloric acid. The precipitated white solids (**P5-COOH**) were collected, dried under the vacuum drying oven, and the next step of the reaction was carried out directly. **P5-COOH** (0.20 g, 0.24 mmol), EDC (0.070 g, 0.36 mmol), HOBt (0.049 g, 0.36 mmol) and LG/DG (0.17 g, 0.27 mmol) were dissolved in 150 mL DCM. Then the mixture was stirred at 25°C for 3 days. After evaporating the

solvents, the remaining solid was dissolved in DCM (20 mL) and poured into a MeOH solution (500 mL). After filtration and dried, the product was purified by recrystallization for four times in THF and MeOH mixture to give the target compounds **P5-DG** (0.269 g, 76.07% yield) and **P5-LG** (0.273 g, 77.21% yield).

P5-DG: ^{1}H NMR (400 MHz, $CDCl_3$/DMSO-d_6 = 1/4, δ, ppm): 8.18-8.13 (m, 1H), 7.22-7.21 (m, 1H), 7.00-6.98 (m, 1H), 6.85-6.77 (m, 10H), 4.28-4.26 (m, 2H), 4.15-4.09 (m, 24H), 3.91-3.83 (m, 3H), 3.76-3.74 (m, 10H), 2.89-2.86 (m, 2H), 2.79-2.76 (m, 2H), 2.49-2.38 (m, 3H), 2.26-2.12 (m, 4H), 2.06-1.88 (m, 2H), 1.24-1.04 (m, 54H), 0.89-0.84 (m, 8H), 0.72-0.62 (m, 4H), 0.23-0.13 (m, 4H).

^{13}C NMR (101 MHz, $CDCl_3$, δ, ppm): 172.76, 172.18, 171.37, 150.46, 150.24, 150.22, 150.19, 150.13, 150.11, 149.46, 128.29, 127.88, 127.83, 127.79, 127.74, 127.68, 114.76, 113.28, 113.19, 113.18, 113.14, 113.00, 79.13, 78.80, 78.47, 68.09, 55.64, 55.55, 55.43, 55.34, 55.31, 53.30, 40.76, 40.56, 40.35, 40.14, 39.93, 39.72, 39.51, 39.27, 32.95, 32.57, 31.81, 29.97, 29.85, 29.76, 29.73, 29.62, 29.57, 29.40, 29.28, 29.27, 29.24, 29.11, 29.08, 29.05, 28.84, 27.81, 26.78, 26.10, 22.60, 14.29.

MALDI-FTICR-MS: calculated for $C_{89}H_{135}N_3O_{13}$, $[M+H]^+$ 1455.0067; found $[M+H]^+$ 1456.0094, $[M+Na]^+$ 1476.9885, $[M+K]^+$ 1492.9622.

P5-LG: ^{1}H NMR (400 MHz, $CDCl_3$/DMSO-d_6 = 1/3, δ, ppm): 8.15-8.11 (m, 1H), 7.61-7.59 (m, 1H), 7.31-7.29 (m, 1H), 6.80-6.78 (m, 10H), 4.20-4.15 (m, 2H), 3.87-3.76 (m, 10H), 3.70-3.61 (m, 27H), 2.97-2.93 (m, 2H), 2.85-2.79 (m, 2H), 2.43-2.34 (m, 3H), 2.13-2.02 (m, 4H), 1.95-1.72 (m, 2H), 1.23-1.01 (m, 54H), 0.88-0.82 (m, 10H), 0.67-0.64 (m, 2H), 0.37-0.34 (m, 4H).

^{13}C NMR (101 MHz, $CDCl_3$, δ, ppm): 172.72, 172.17, 171.37, 150.48, 150.28, 150.24, 150.21, 150.17, 150.16, 149.50, 128.31, 127.92, 127.87, 127.83, 127.79, 127.78, 114.81, 113.36, 113.25, 113.20, 113.08, 113.02, 79.11, 78.79, 78.46, 68.11, 55.67, 55.58, 55.46, 55.38, 55.33, 53.29, 40.78, 40.58, 40.37, 40.16, 39.95, 39.74, 39.53, 39.27, 32.94, 32.56, 31.80, 29.93, 29.82, 29.74, 29.71, 29.67, 29.60, 29.56, 29.55, 29.38, 29.26, 29.25, 29.09, 28.97, 28.86, 27.86, 26.78, 26.15, 22.58, 14.26.

MALDI-FTICR-MS: calculated for $C_{89}H_{135}N_3O_{13}$, $[M]^+$ 1453.9989; found $[M]^+$ 1453.9985, $[M+H]^+$ 1455.0056, $[M+Na]^+$ 1476.9885, $[M+K]^+$ 1492.9622.

1.4 Supplementary NMR and MS spectra

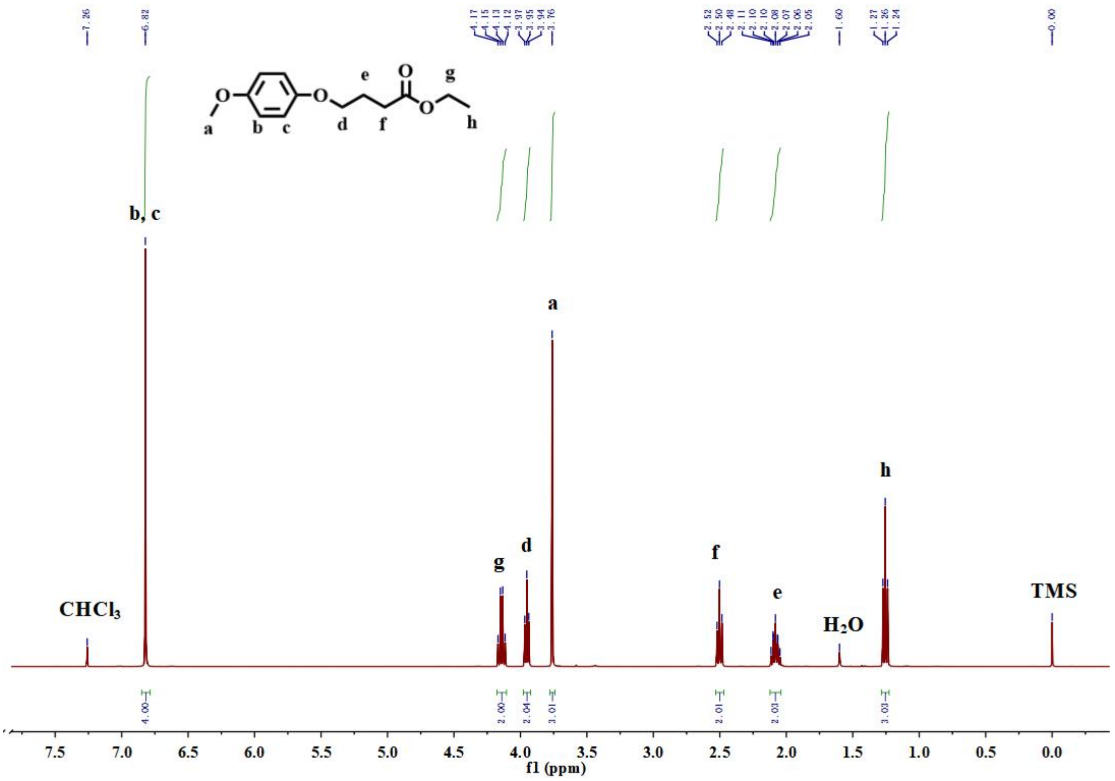

Figure S1 ^{1}H-NMR spectrum of molecule **1** in $CDCl_3$.

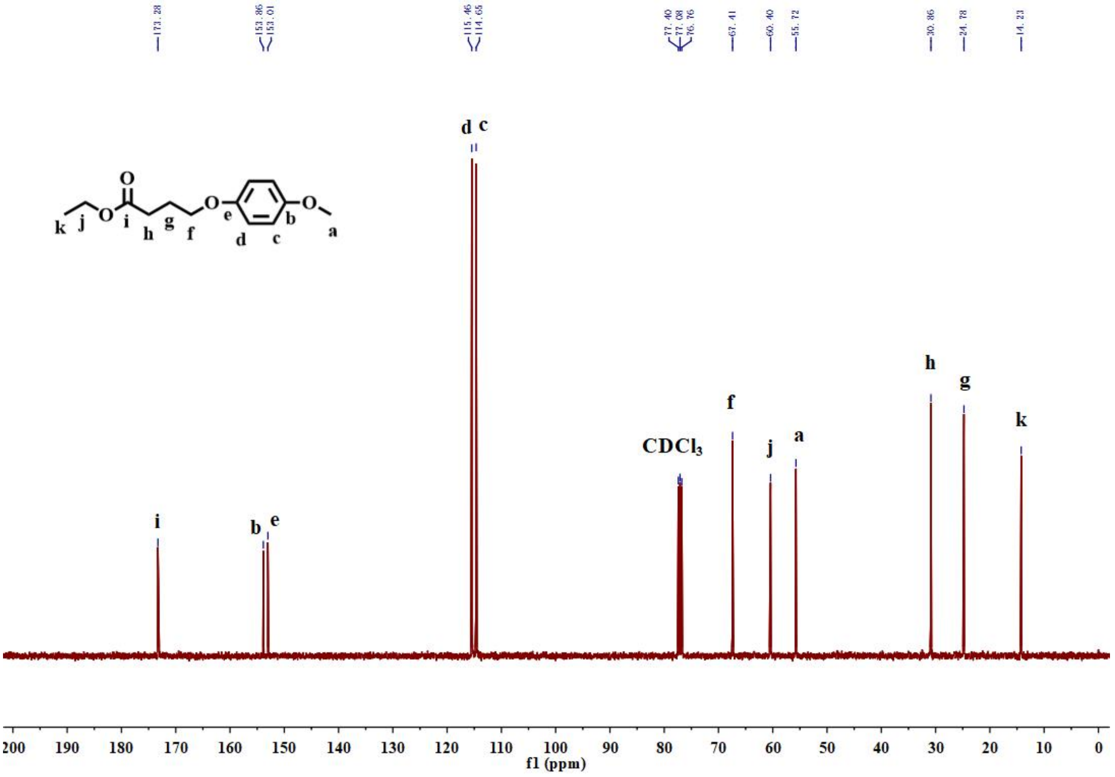

Figure S2 ^{13}C-NMR spectrum of molecule **1** in $CDCl_3$.

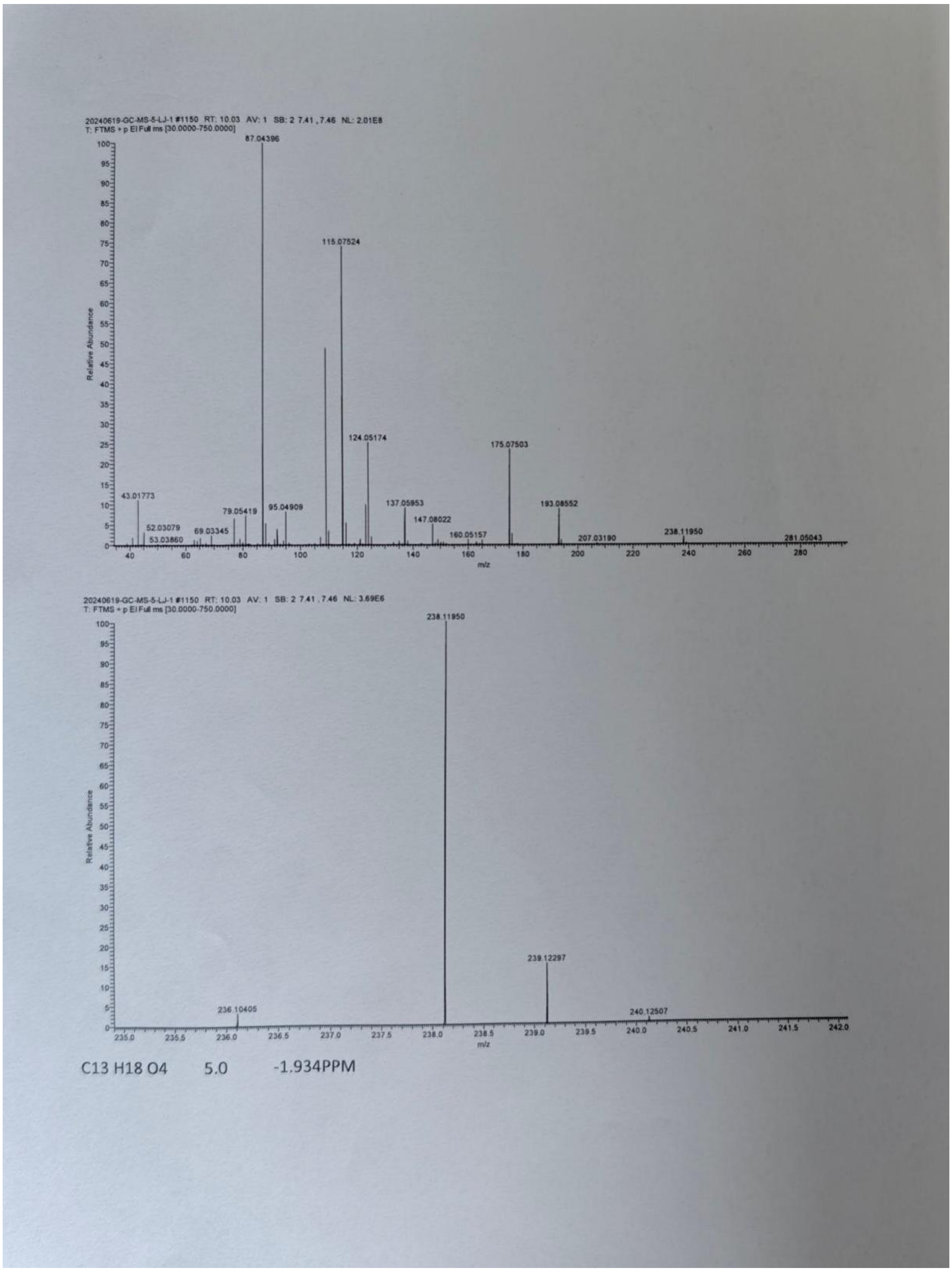

Figure S3 EI-MS spectra of molecule **1**.

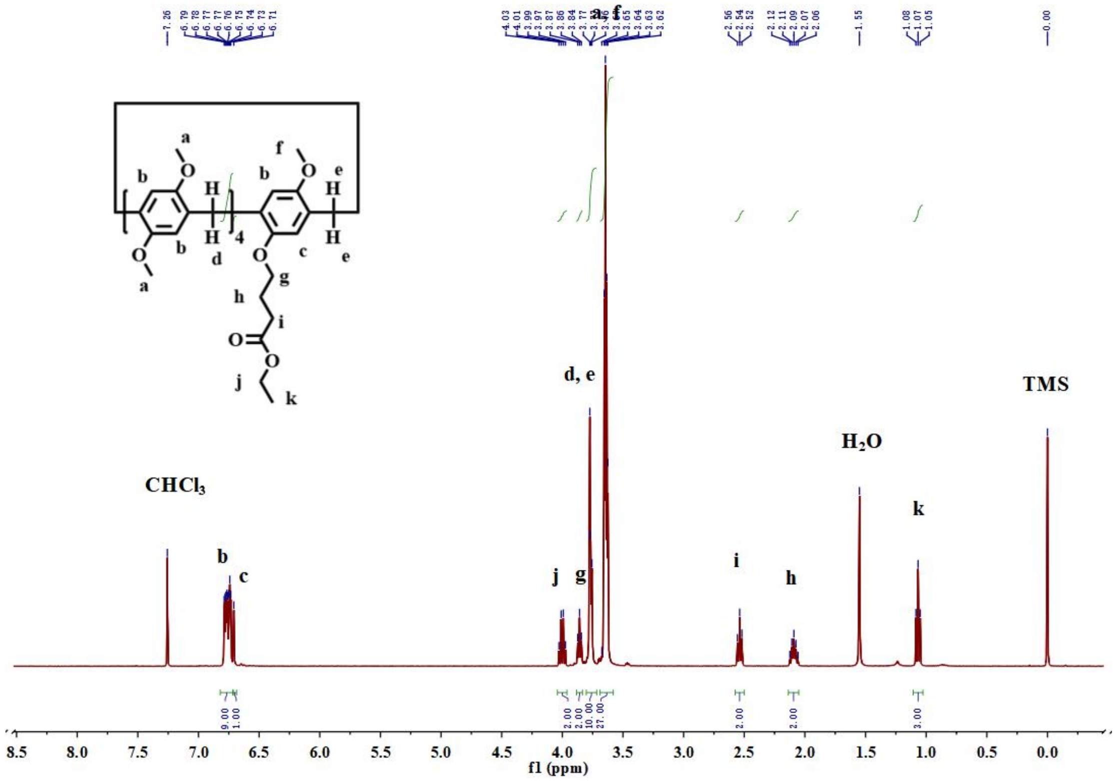

Figure S4 ^{1}H-NMR spectrum of molecule **P5-COOEt** in $CDCl_3$.

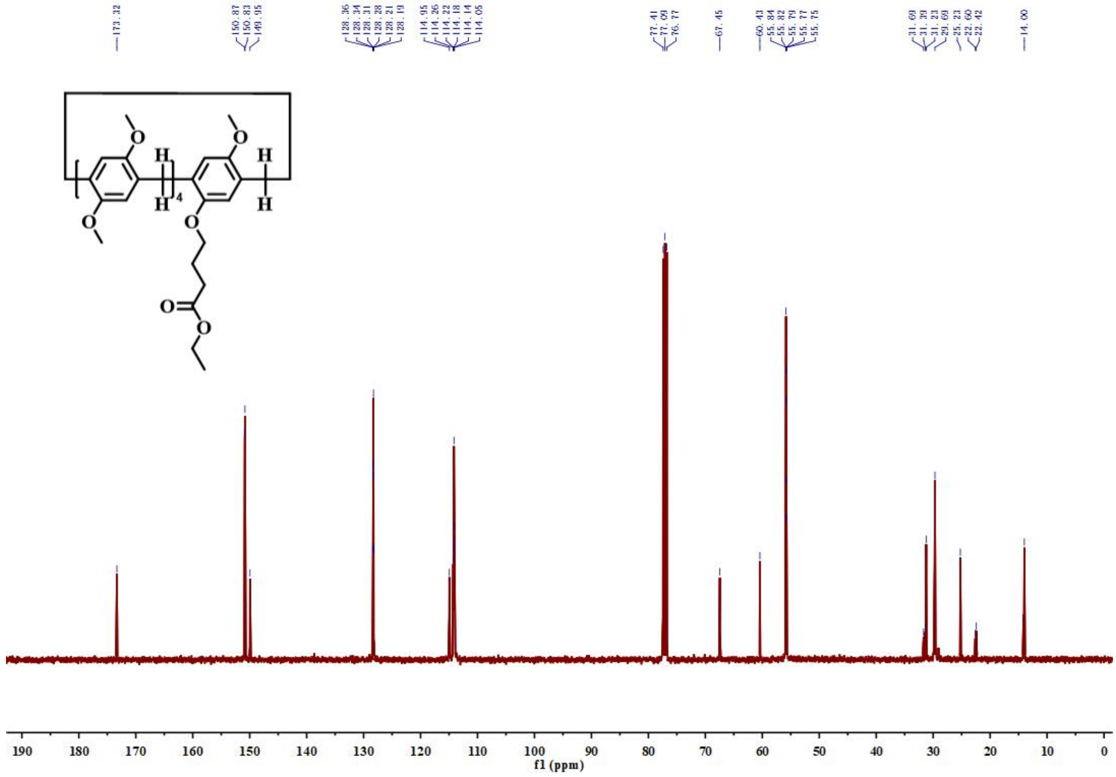

Figure S5 ^{13}C-NMR spectrum of molecule **P5-COOEt** in $CDCl_3$.

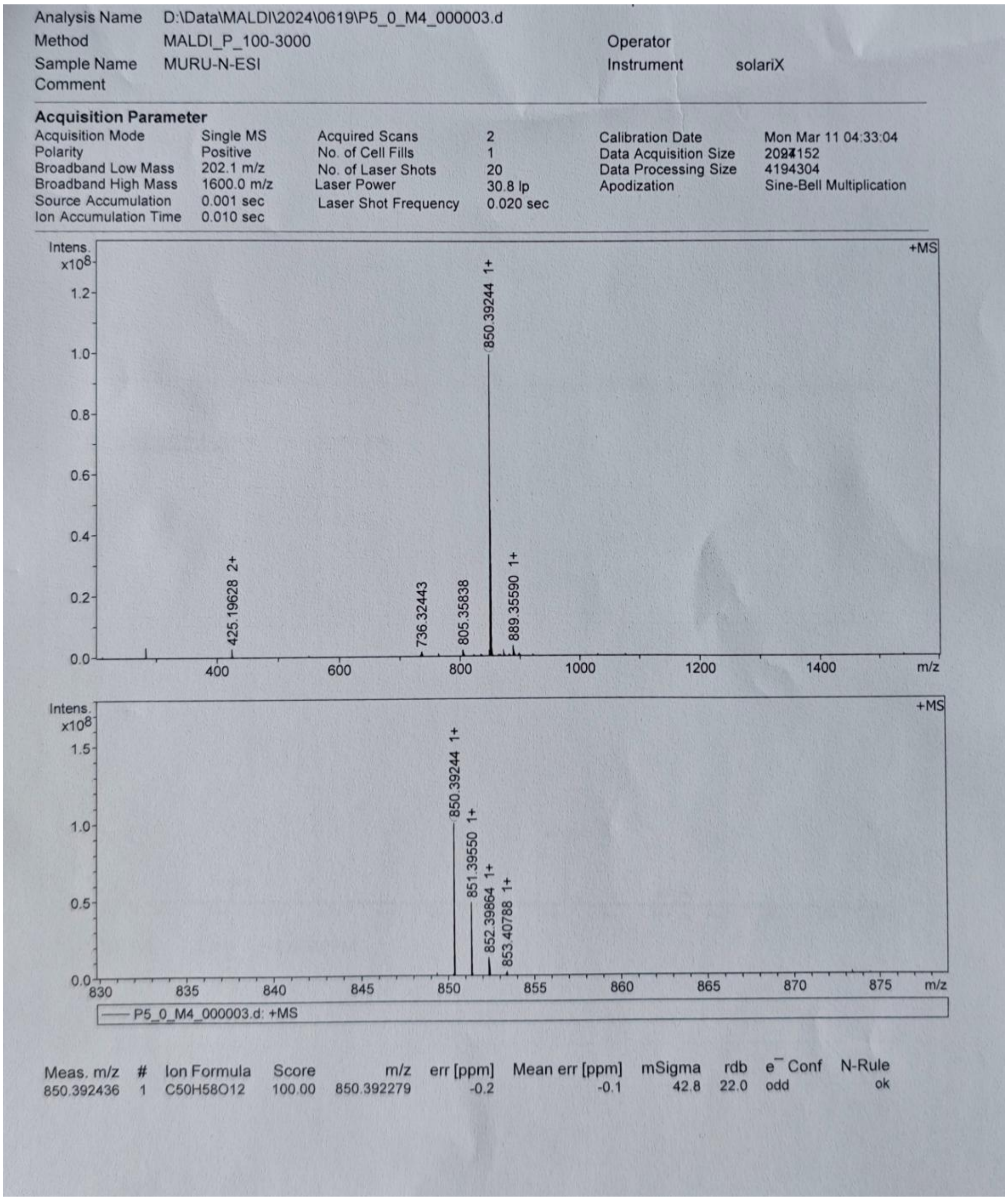

Figure S6 MALDI-FTICR-Mass spectra of molecule **P5-COOEt**.

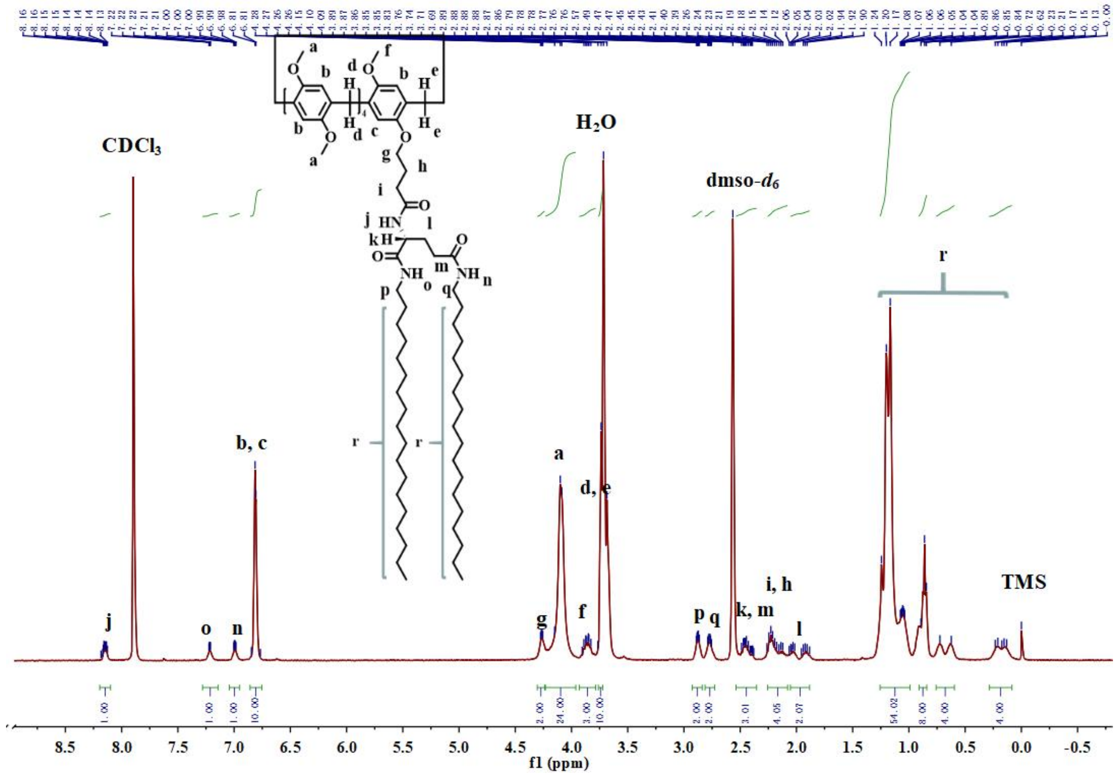

Figure S7 ^{1}H-NMR spectrum of molecule **P5-DG** in $CDCl_3$/DMSO-d_6 = 1/4.

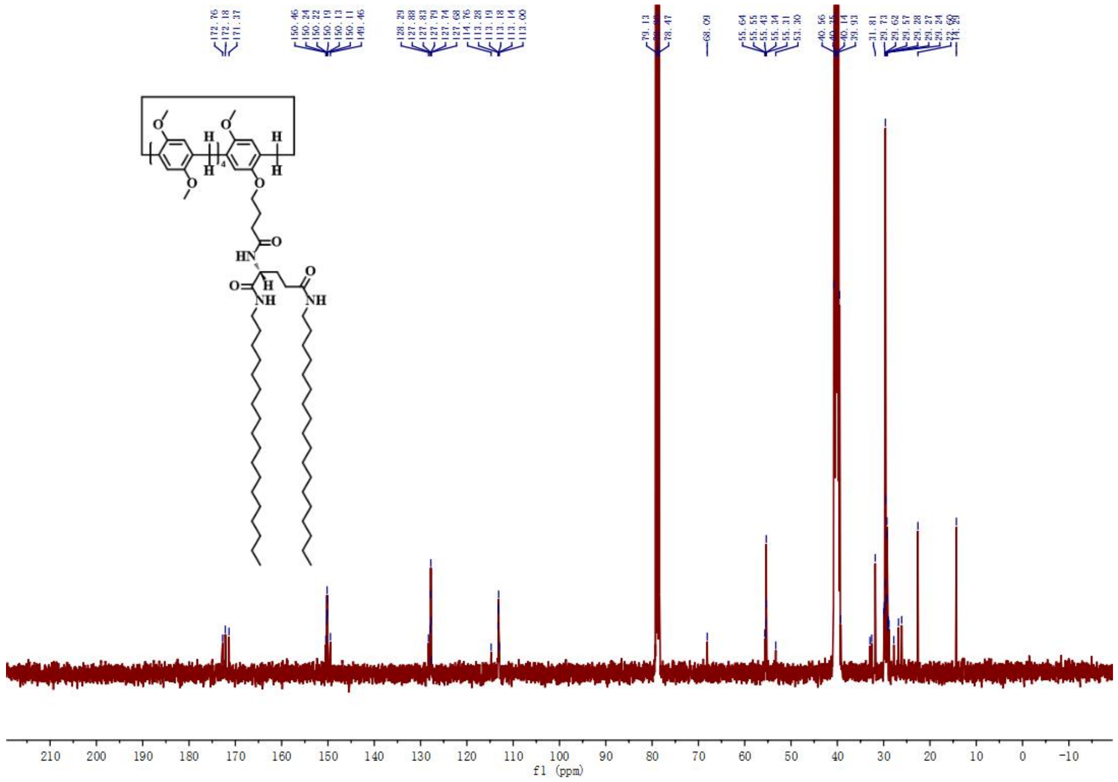

Figure S8 ^{13}C-NMR spectrum of molecule **P5-DG** in $CDCl_3$/DMSO-d_6 = 1/1.

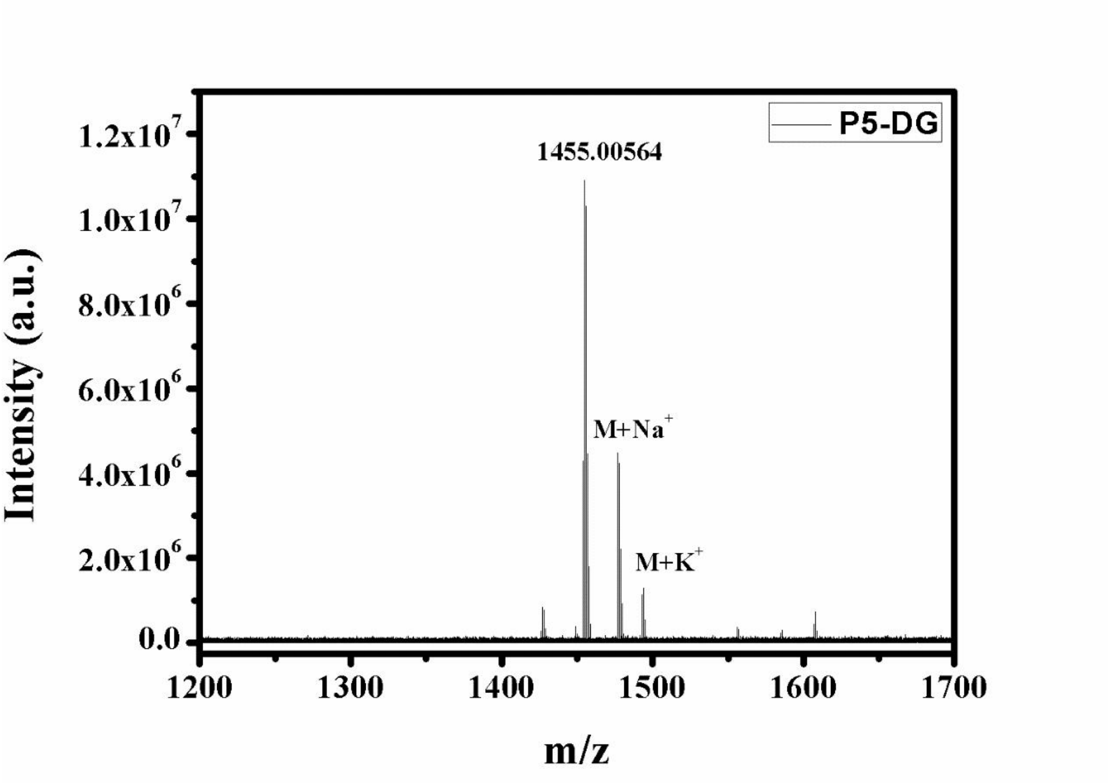

Figure S9 MALDI-FTICR-Mass spectra of molecule **P5-DG**.

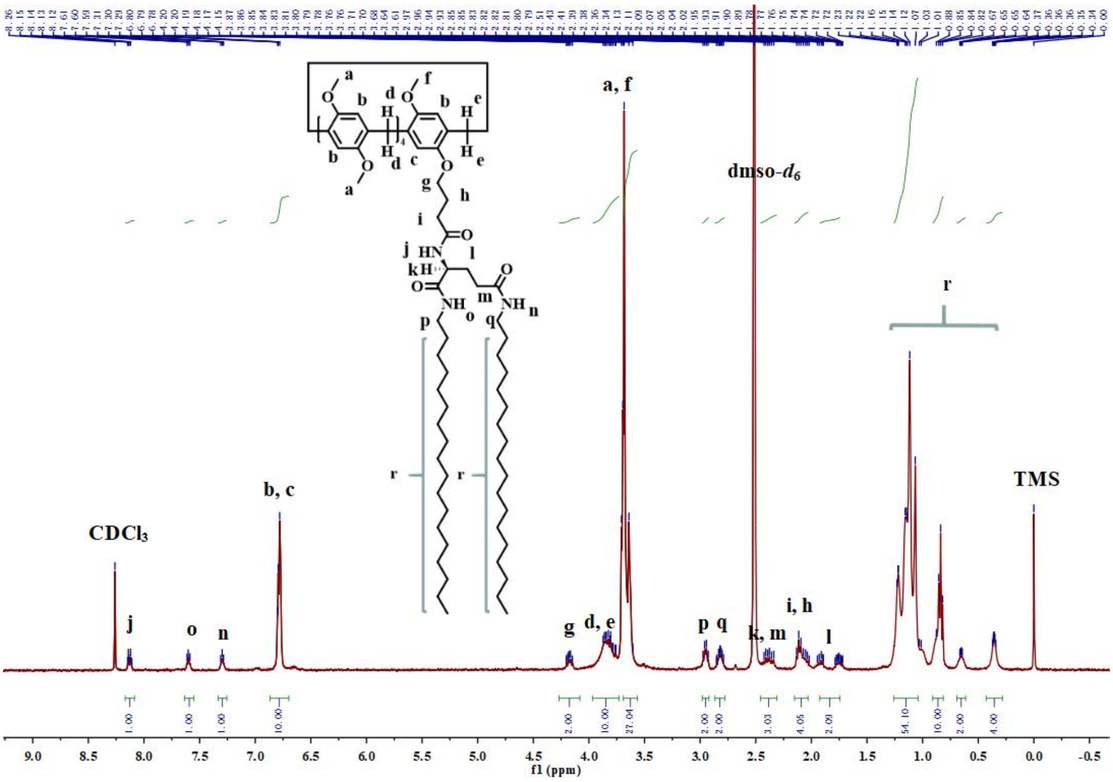

Figure S10 ^{1}H-NMR spectrum of molecule **P5-LG** in $CDCl_3$/DMSO-d_6 = 1/3.

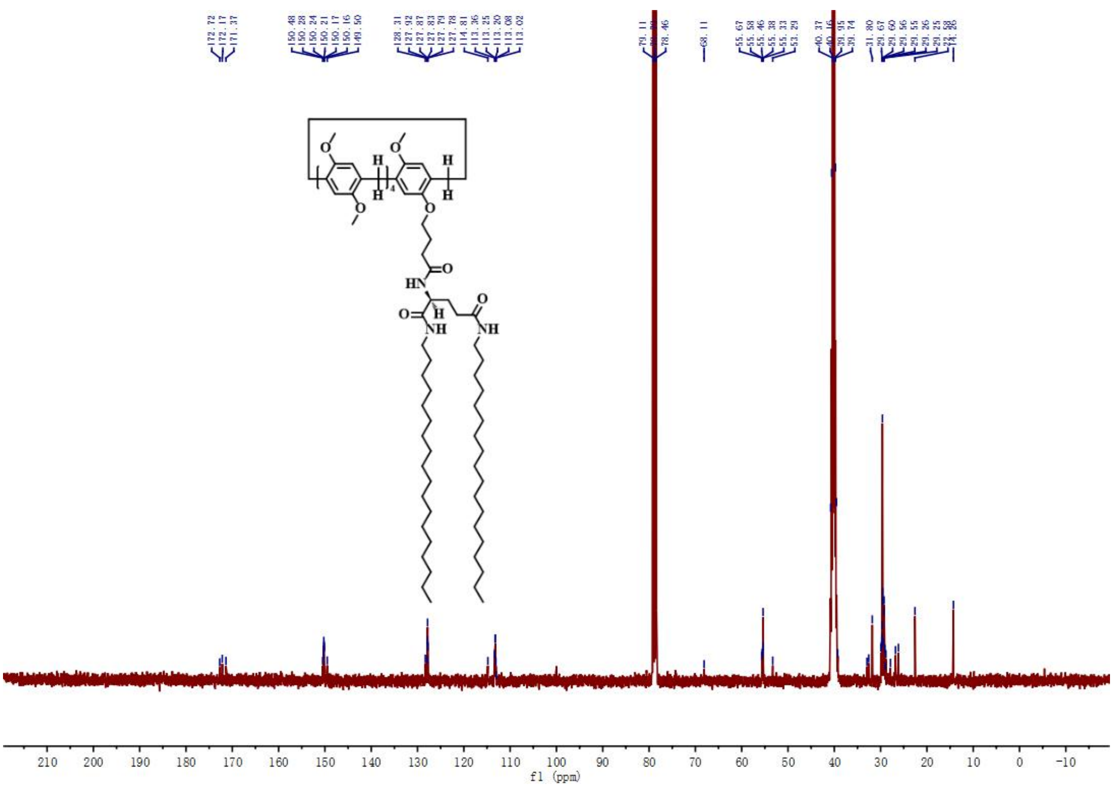

Figure S11 ^{13}C-NMR spectrum of molecule **P5-LG** in $CDCl_3$/DMSO-d_6 = 1/1.

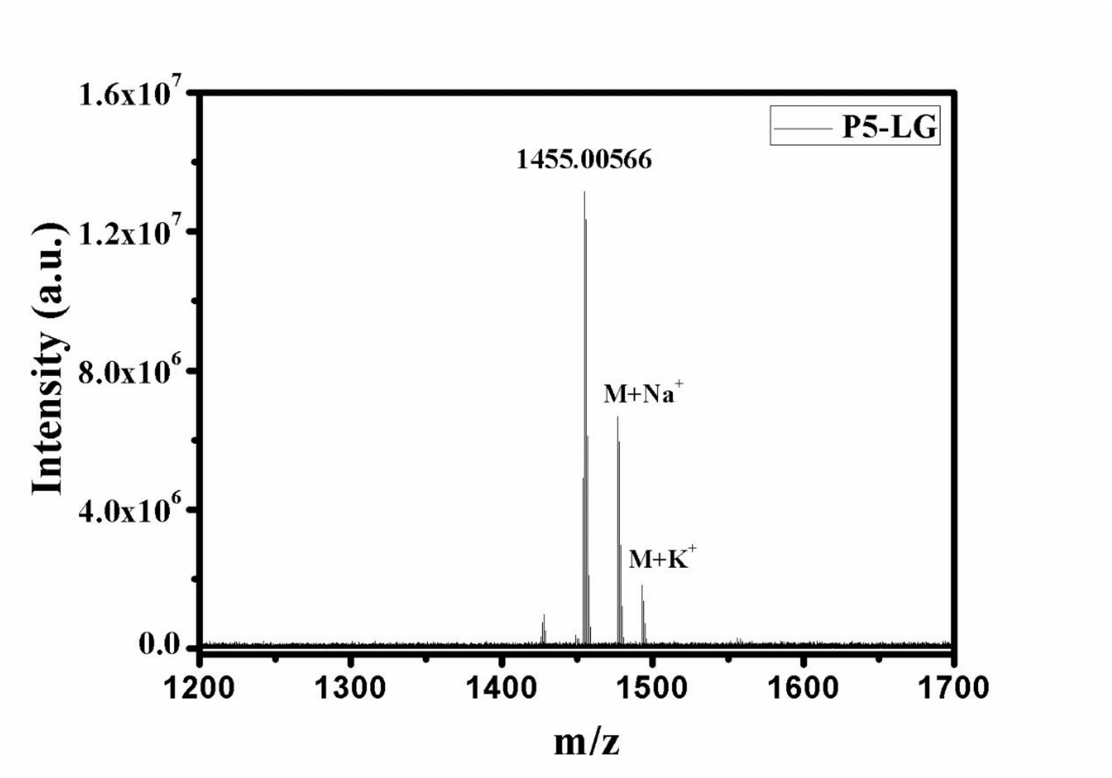

Figure S12 MALDI-FTICR-Mass spectra of molecule **P5-LG**.

1.5 Sample Preparations

Self-assembly protocol of P5 in THF/H_2O (v/v = 6/4)

Typically, **P5** (0.22 mg, 0.15 μmol) were dissolved in 300 μL tetrahydrofuran. Then quickly add 200 μL H_2O (anti-solvent) at one time. The vesicle was prepared by drop-casting the THF/H_2O solution of **P5** on the quartz wafer or silicon wafer.

Self-assembly protocol of P5 in THF/H_2O (v/v = 3/7)

Typically, **P5** (0.22 mg, 0.15 μmol) were dissolved in 150 μL tetrahydrofuran. Then quickly add 350 μL H_2O (anti-solvent) at one time. The Nanoscale Toroidal was prepared by drop-casting the THF/H_2O solution of **P5** on the quartz wafer or silicon wafer.

Self-assembly protocol of P5 in THF/H_2O (v/v = 1/9)

Typically, **P5** (0.22 mg, 0.15 μmol) were dissolved in 50 μL tetrahydrofuran. Then quickly add 450 μL H_2O (anti-solvent) at one time. The spherical aggregates was prepared by drop-casting the THF/H_2O solution of **P5** on the quartz wafer or silicon wafer.

1.6 Theoretical simulation

1.6.1 Structure optimization of single molecule

The complex was subjected to rigorous Density Functional Theory (DFT) calculations with a focus on molecular optimization. All atoms were treated using the B3LYP-D3(BJ)/def2svp method. To further elucidate its properties, Time-Dependent Density Functional Theory (TD-DFT) calculations were performed at the same level of theory using the Gaussian16[S3] software package.

1.6.2 Structure optimization (CP2K) and IRI weak interaction description

Geometry optimizations were performed using the GFN1-xTB[S4] tight-binding quantum chemical method, augmented by the orbital transformation (OT)[S5] technique. Single-point calculations employed the PBE-D3(BJ)/pob-TZVP functional with OT, both implemented in CP2K[S6] (version 2023.1). Non-covalent interactions (NCIs) were analyzed using the Interaction Region Indicator (IRI)[S7] as implemented in Multiwfn 3.8 (dev)[S8]. Visualizations were generated using VMD[S9].

2 Results and Discussion

2.1 Supplementary spectra of P5-DG/LG in different solvents

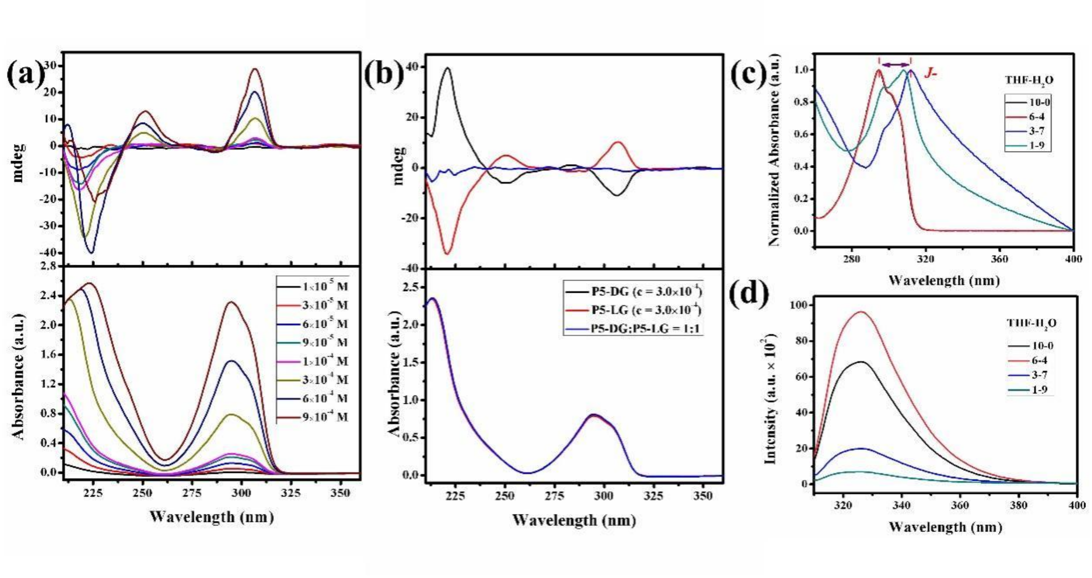

Figure S13 (a) CD spectra (top) and UV–vis absorption (Abs) (bottom) of different concentrations of **P5-LG** in THF, and the cuvette path length is 1 mm, (b) CD spectra (top) and UV–vis absorption (Abs) (bottom) of **P5-DG**, **P5-LG** and **P5-DG/P5-LG** (c/c = 1/1) in THF, and the cuvette path length is 1 mm. (c) Abs and (d) Emission (excited at 295 nm, Ex bandwidth: 5 nm; Em bandwidth: 5 nm) spectra of **P5-LG** in different solvents at 48 h.

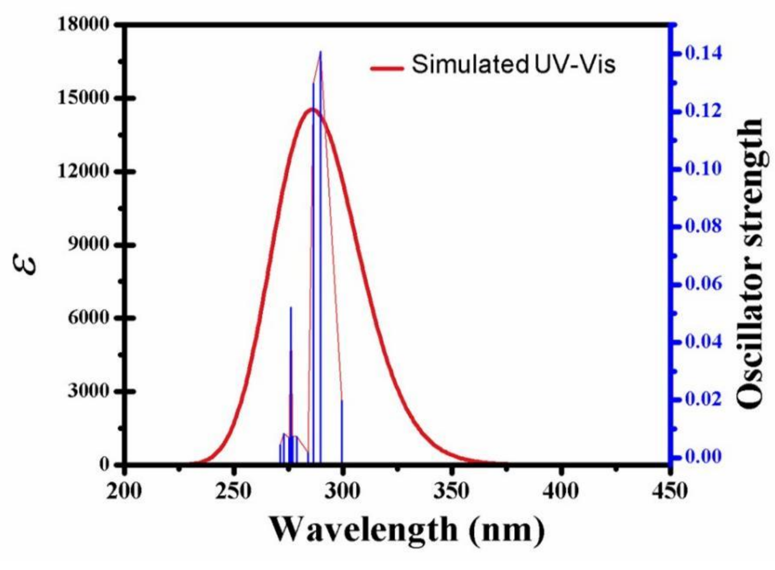

Figure S14 Simulated UV–vis absorption (Abs) of **P5-LG.**

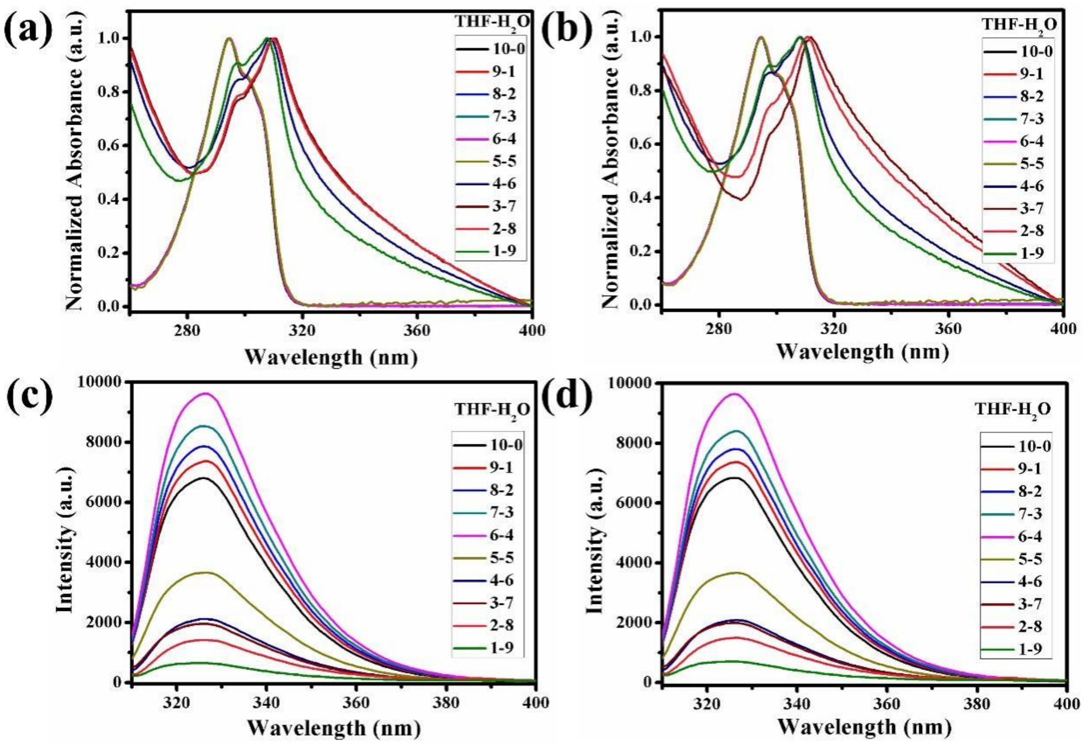

Figure S15 UV–vis absorption (Abs) of (a) **P5-DG** and (b) **P5-LG** and Emission (excited at 295 nm, Ex bandwidth: 5 nm; Em bandwidth: 5 nm) spectra of (c) **P5-DG** and (d) **P5-LG** in different solvents at 48 h.

2.2 Supplementary photographs and DLS image of P5-DG/LG in different solvents

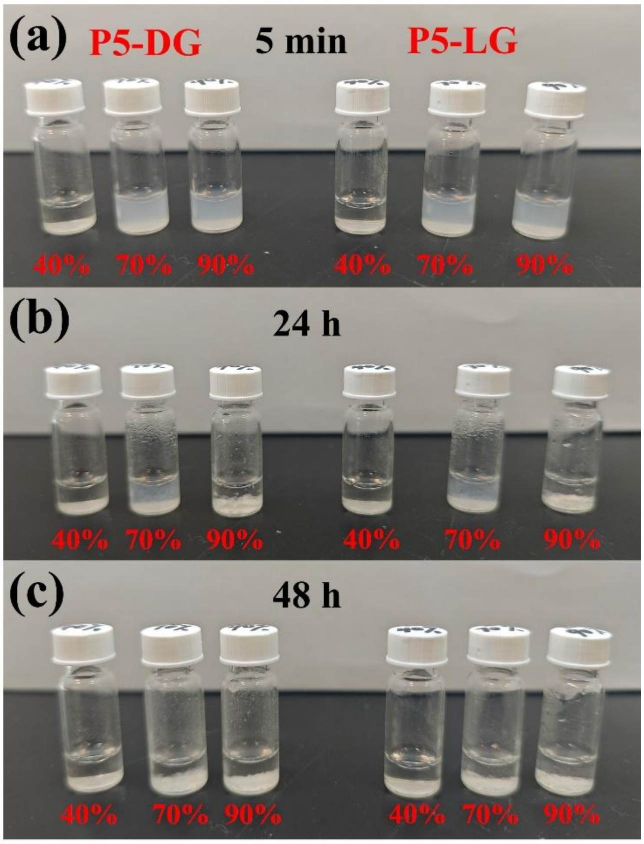

Figure S16 Photographs of **P5-DG** and **P5-LG** (0.03 mM) in solvents with different polarity at (a) 5 min, (b) 24 h and (c) 48 h.

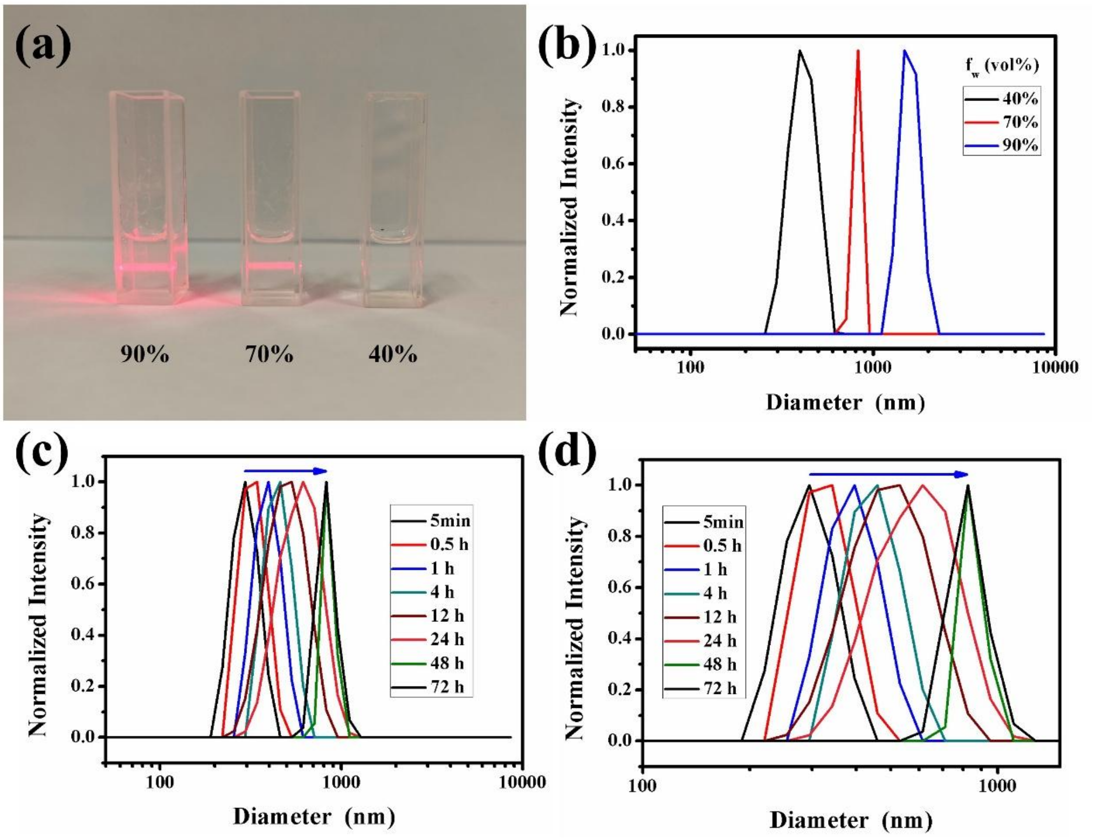

Figure S17 (a) Photograph of the Tyndall effect of **P5-DG** under a beam of red light, (b) DLS data of **P5-DG** in THF/H_2O at 293 K, (c) The time-dependent DLS data of **P5-DG** in THF/H_2O (v/v = 3/7) at 293 K, (d) Partial enlarged view of (c).

2.3 Supplementary SEM and TEM image of P5-DG/LG in different solvents

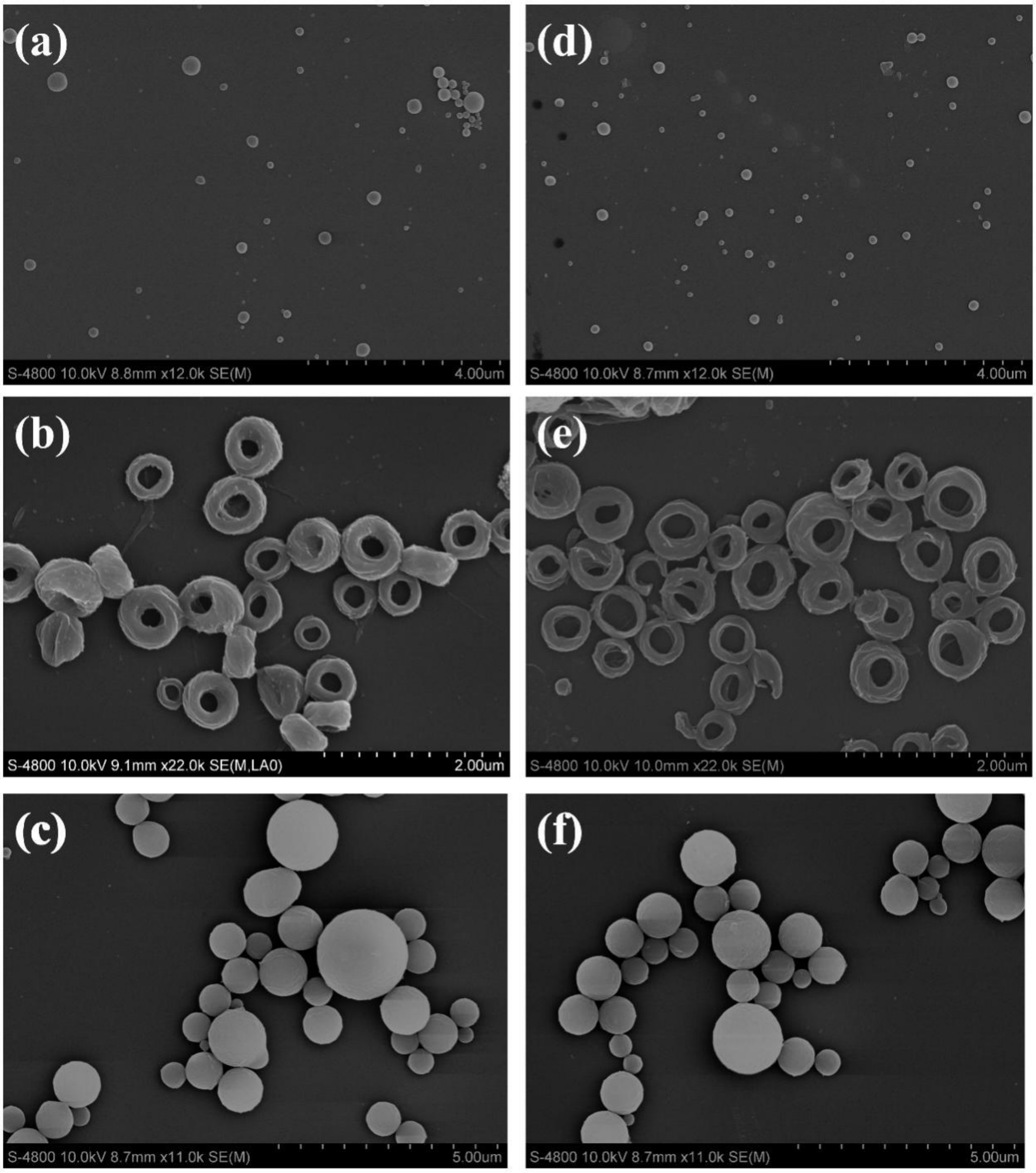

Figure S18 SEM images of **P5-DG** in (a) THF/H_2O (v/v=6/4), (b) THF/H_2O (v/v=3/7), (c) THF/H_2O (v/v=1/9) and **P5-LG** in (d) THF/H_2O (v/v=6/4), (e) THF/H_2O (v/v=3/7), (f) THF/H_2O (v/v=1/9) for 48 hours at 25 ℃.

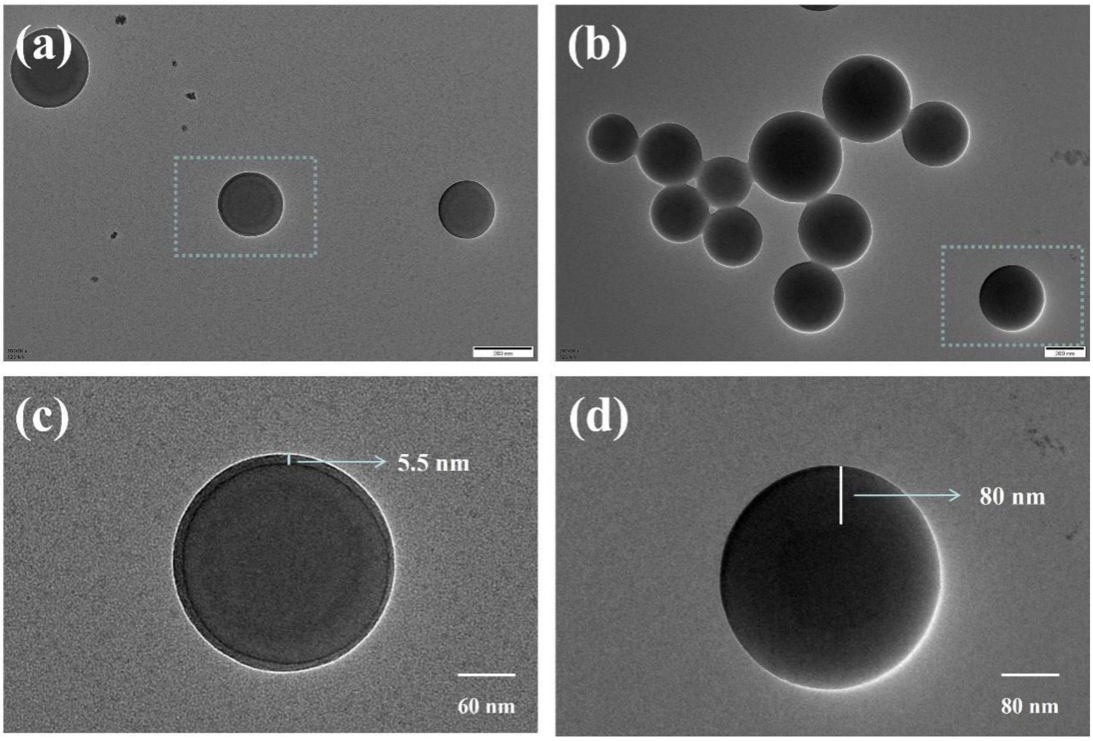

Figure S19 (a) TEM images of **P5-DG** in THF/H_2O (v/v=6/4) for 48 h at 25 ℃, (b) TEM images of **P5-DG** in THF/H_2O (v/v=3/7) for 5 min at 25 ℃, (c) enlarged image of part (a), (d) enlarged image of part (b).

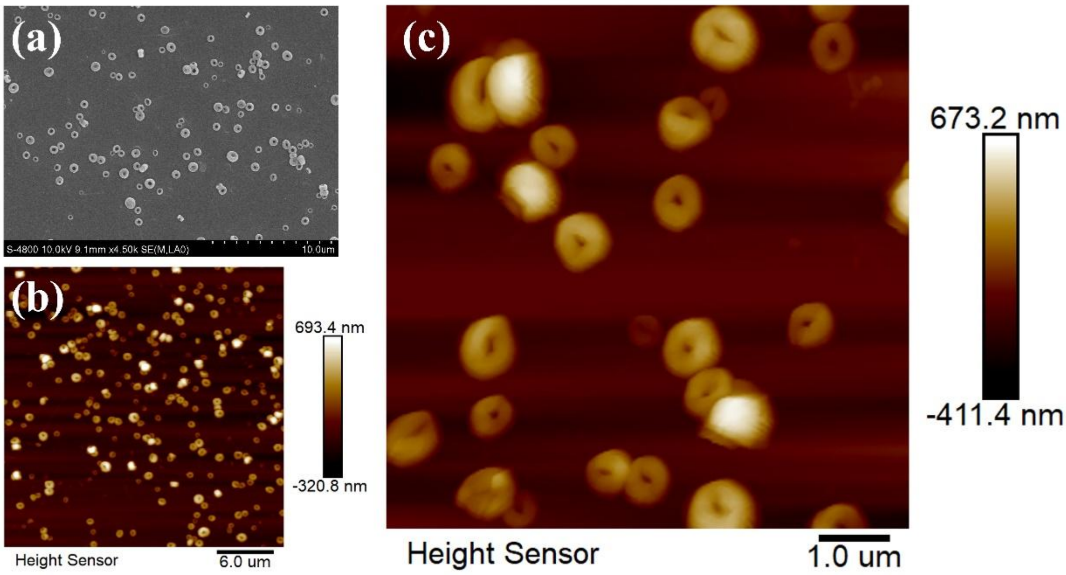

Figure S20 (a) Large-scale SEM image of the topologically macrocycle in **P5-DG** following 12-months aging in THF/H_2O (v/v=3/7), (b) and (c) AFM image of **P5-DG** following 12-months aging in THF/H_2O (v/v=3/7).

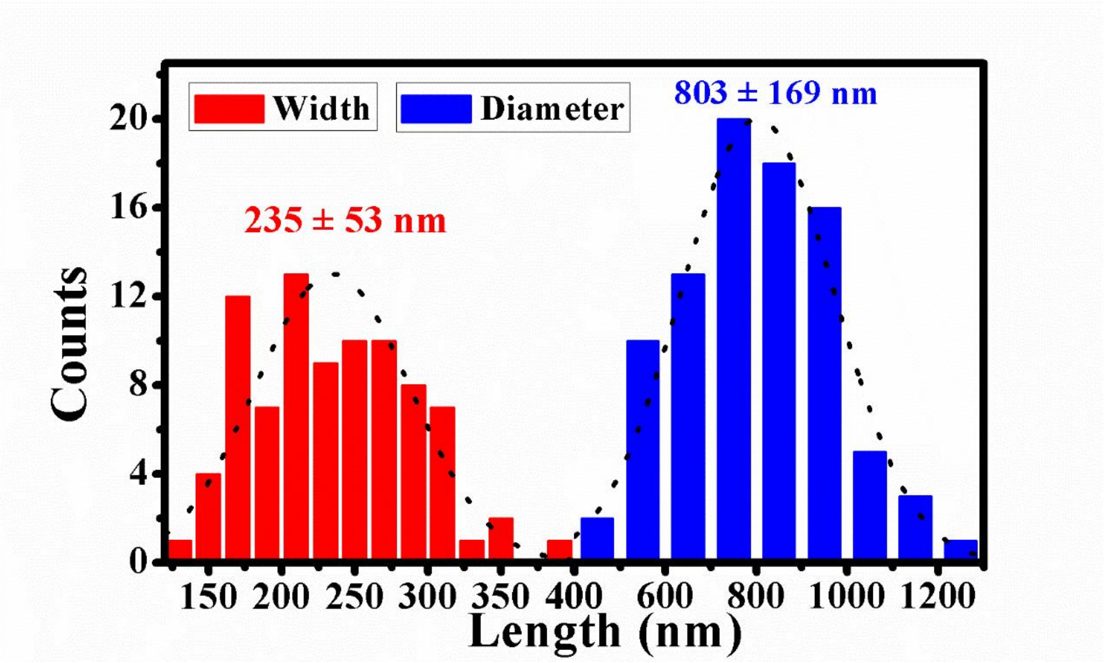

Figure S21 Statistical analysis of toroidal width and diameter of **P5-LG**.

2.4 Supplementary CD and CPL spectra of P5-DG/LG

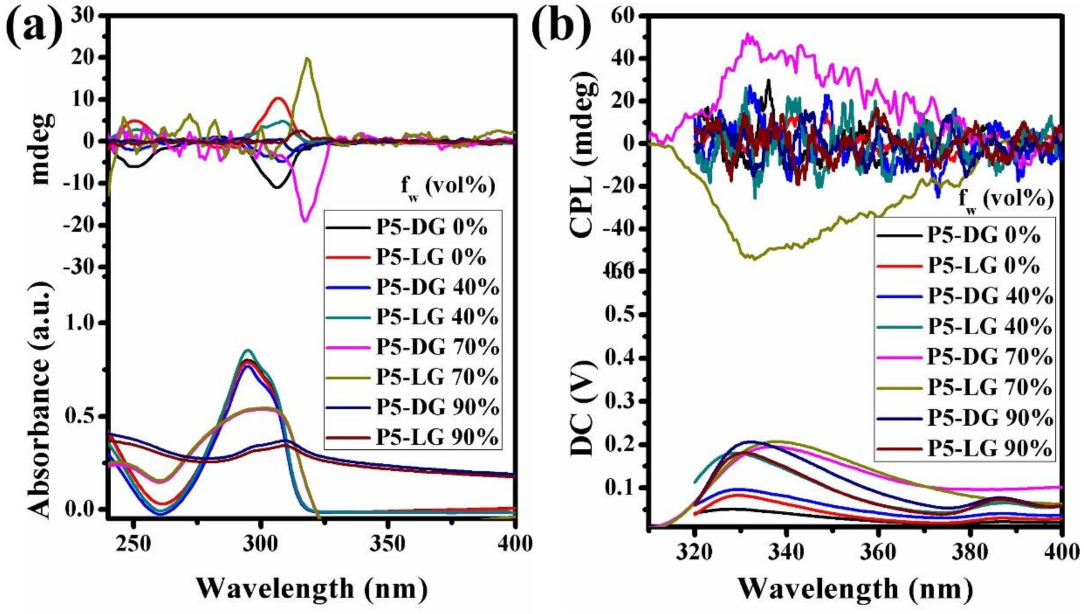

Figure S22 (a) CD spectra (top) and UV–vis absorption (Abs) (bottom) of **P5-DG/LG** corresponding to CD spectra, (b) CPL (Ex = 285 nm) spectra (top) and DC value (bottom) of **P5-DG/LG** corresponding to CPL spectra.

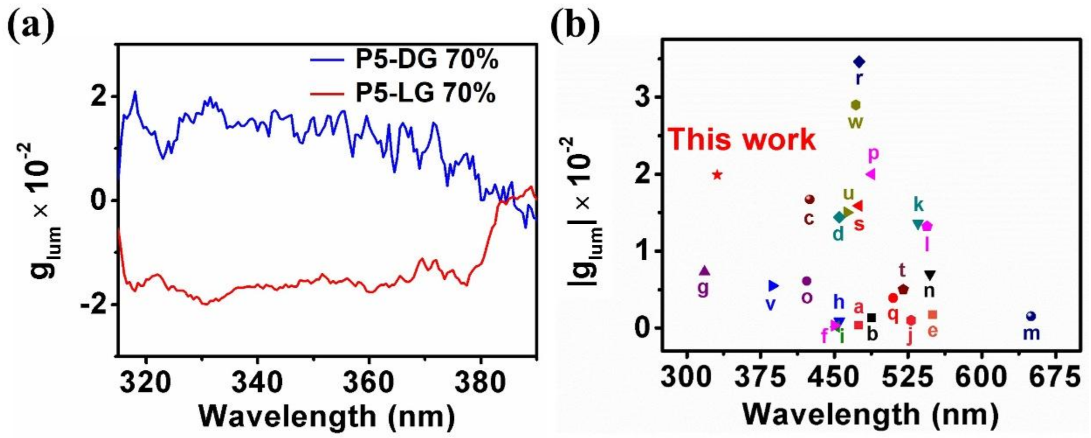

Figure S23 (a) The g_{lum} value spectra of CPL corresponding to Fig. 3b, (b) The $|g_{lum}|$ values of CPL-active pillar[5]arenes reported previously. (a-w represent references 3-5, 14, 18, 19, 32, and 35-50, respectively. For the sake of convenience in statistical analysis, the emission wavelengths corresponding to the g-values in the references 4 and 45-47 are taken as the median values.)

Table S1. The $|g_{lum}|$ values of CPL-active pillar[5]arenes reported previously

DOI	*Journal Name*	λ_{ex}	Em	$\|g_{lum}\|_{max}$	Ref
10.1002/anie.202502381.	*Angew Chem Int Ed.*	245 nm	433 nm	3.4×10^{-4}	3
10.1002/adom.202202913.	*Adv. Optical Mater.*	340 nm	470 - 490 nm	1.28×10^{-3}	4
10.1038/s41467-025-57461-x	*Nat Commun.*	305 nm	412 nm	1.67×10^{-2}	5
10.1038/s41467-024-54961-0	*Nat Commun.*	350 nm	~ 440 nm	1.9×10^{-2}	14
10.1039/d2sc00952h	*Chem Sci.*	385 nm	550 nm	1.7×10^{-3}	18
10.1002/chem.202500771	*Chem Eur J.*	306 nm	475 nm	3.25×10^{-4}	19
10.1002/anie.202209222	*Angew Chem Int Ed.*	240 nm	318 nm	7.3×10^{-3}	32
10.1002/anie.202001145	*Angew Chem Int Ed.*	303 nm	455 nm	8.78×10^{-4}	35
10.1021/acs.joc.1c01175	*J Org Chem.*	298 nm	452 nm	1.93×10^{-4}	36
10.1002/chem.202100458	*Chem Eur J.*	330 nm	528 nm	1.0×10^{-3}	37
10.1002/anie.202100934	*Angew Chem Int Ed.*	360 nm	535 nm	1.36×10^{-2}	38
10.31635/ccschem.022.202101749	*CCS Chem.*	300 nm	520 nm	1.32×10^{-2}	39
10.1021/acs.orglett.2c00313	*Org Lett.*	373 nm	650 nm	1.51×10^{-3}	40
10.1039/d2sc04168e	*Chem Sci.*	280 nm	547 nm	8.4×10^{-3}	41
10.1039/d2sc06000k	*Chem Sci.*	305 nm	422 nm	6.1×10^{-3}	42

10.1002/anie.202302978	*Angew Chem Int Ed.*	330 nm	515 nm	2.0×10^{-2}	43
10.1002/advs.202305149	*Adv Sci.*	298 nm	510 nm	3.91×10^{-3}	44
10.1002/anie.202319502	*Angew Chem Int Ed.*	365 nm	450-500 nm	3.46×10^{-2}	45
10.1007/s11426-024-2197-1	*Sci China Chem.*	365 nm	450-500 nm	1.59×10^{-2}	46
10.1002/anie.202415190	*Angew Chem Int Ed.*	340 nm	430-590 nm	5.0×10^{-3}	47
10.1002/anie.202412548	*Angew Chem Int Ed.*	343 nm	463 nm	1.5×10^{-2}	48
10.1007/s11426-024-2489-3	*Sci China Chem.*	293 nm	387 nm	5.5×10^{-3}	49
10.1002/agt2.482	Aggregate.	250 nm	472 nm	2.9×10^{-2}	50

[a] This panel corresponds to the content of Fig. S23b. (a-w represent references 3-5, 14, 18, 19, 32, and 35-50, respectively.)

2.5 Supplementary IR spectra of P5-DG/LG

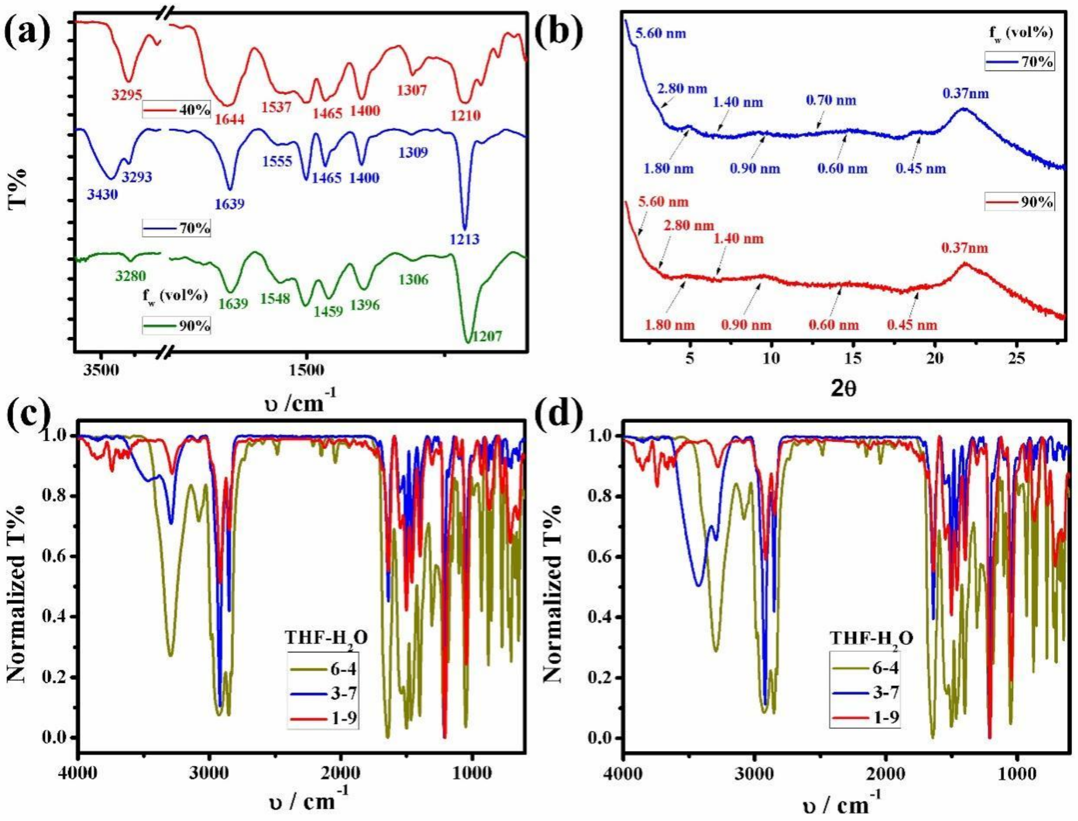

Figure S24 (a) Partial FT-IR spectra of part of **P5-LG** from different volume ratios of THF/H_2O solutions, (b) XRD patterns of **P5-LG** assembly in THF/H_2O (v/v = 1/9, red line) and THF/H_2O (v/v = 3/7, blue line), FT-IR spectra of (c) **P5-DG** and (d) **P5-LG**

from different volume ratios of THF/H_2O solutions.

2.6 Supplementary Theoretical simulation of P5-DG/LG

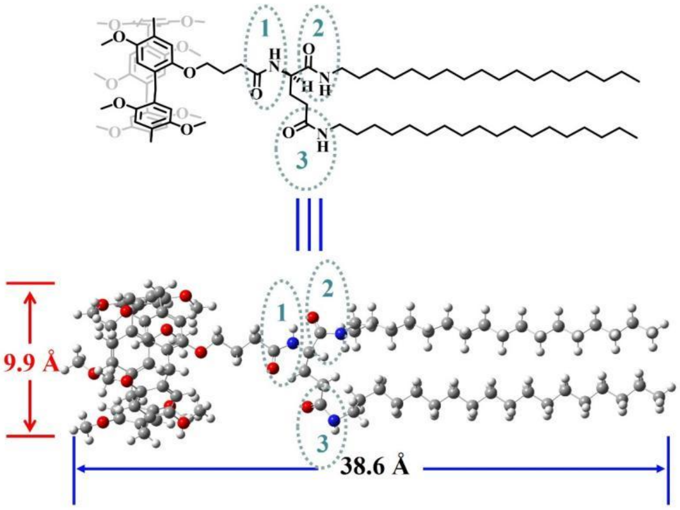

Figure S25 The molecular length and height of **P5-DG** simulated by DFT at the lowest energy.

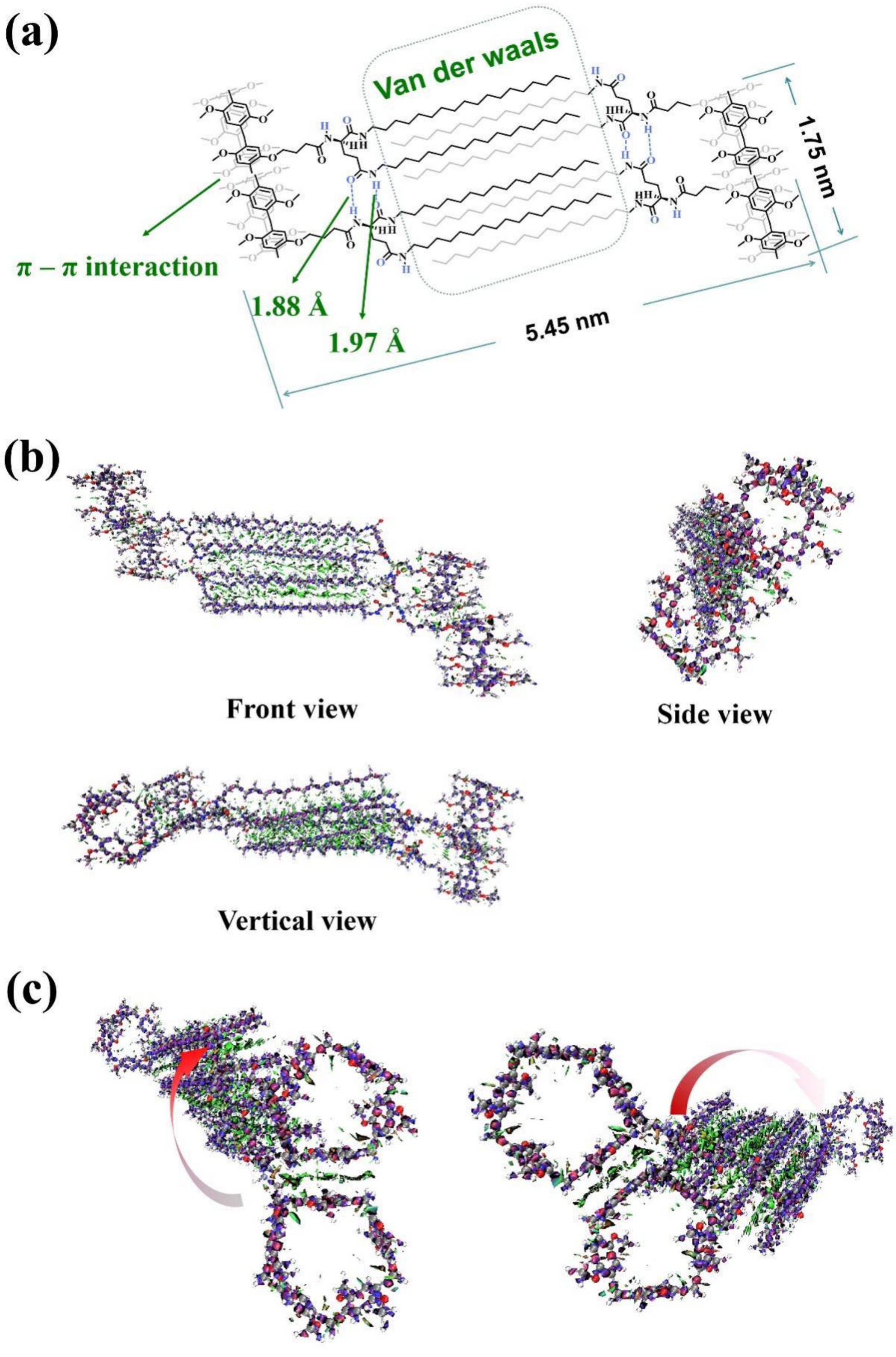

Figure S26 Simulation results of **P5-DG** assembly conformations from five perspectives.

2.7 Investigation of Host-Guest Interactions Between Pillar[5]arene Macrocycles and Long Alkyl Chains

Due to the poor solubility of the molecules in THF/H_2O (v/v = 3/7), which was insufficient for NMR characterization, we employed THF-d_8/D_2O (v/v = 6/4) as the solvent for testing.

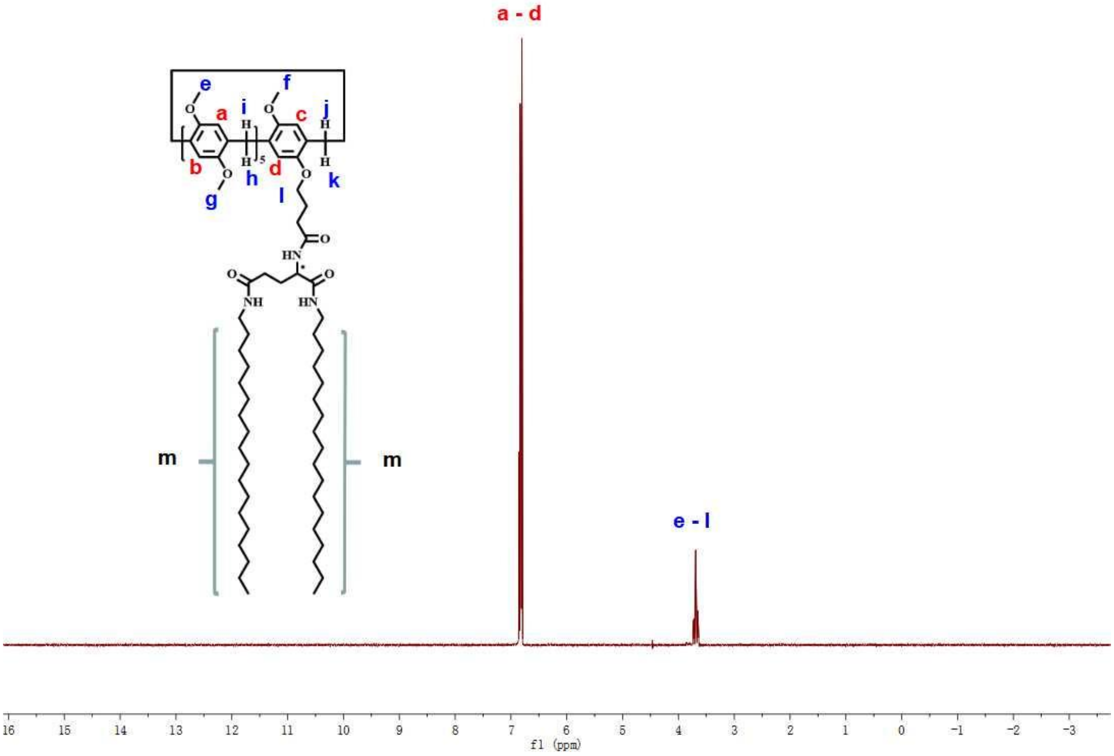

Figure S27 1D-selective gradient NOESY spectrum of molecule **P5-DG** (0.15 μmol) in THF-d_8/D_2O (v/v = 6/4).

2.8 Supplementary SEM and spectra of P5/dyes co-assemblies

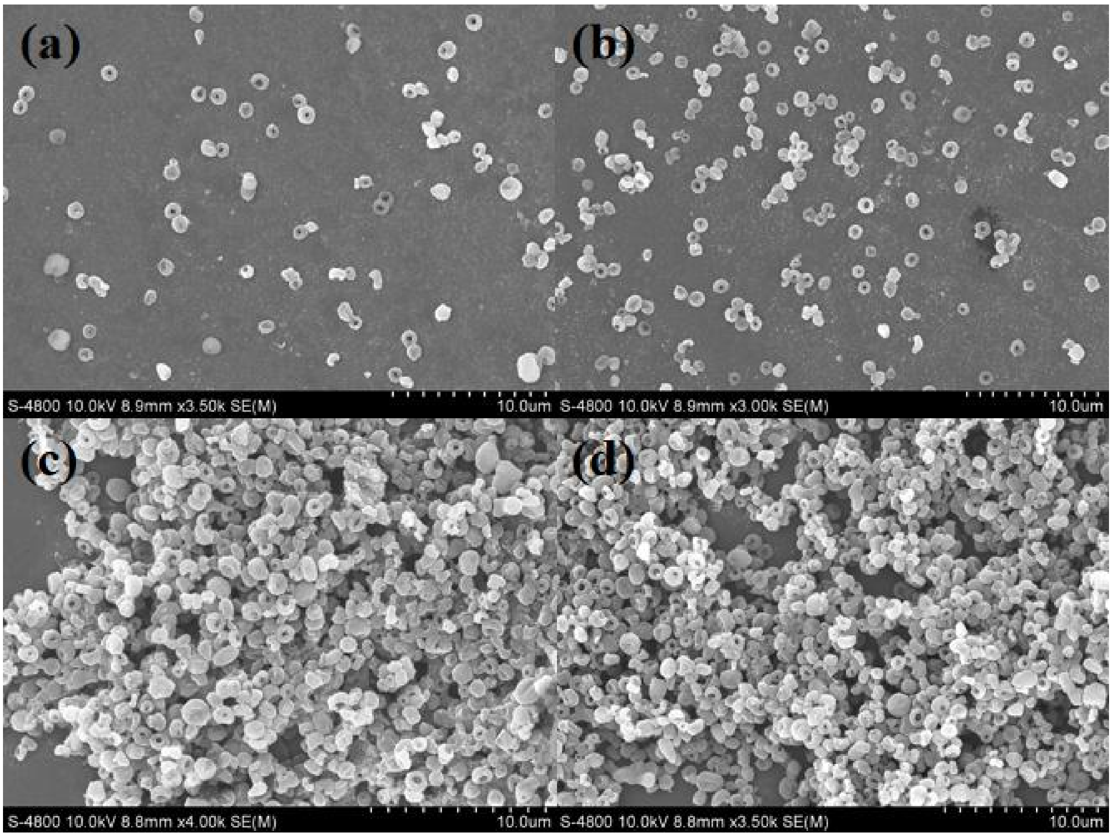

Figure S28 SEM images of (a) **P5-DG+TPPS**, (b) **P5-LG+TPPS**, (c) **P5-DG+CBS** and (d) **P5-LG+CBS** in THF/H_2O (v/v=3/7).

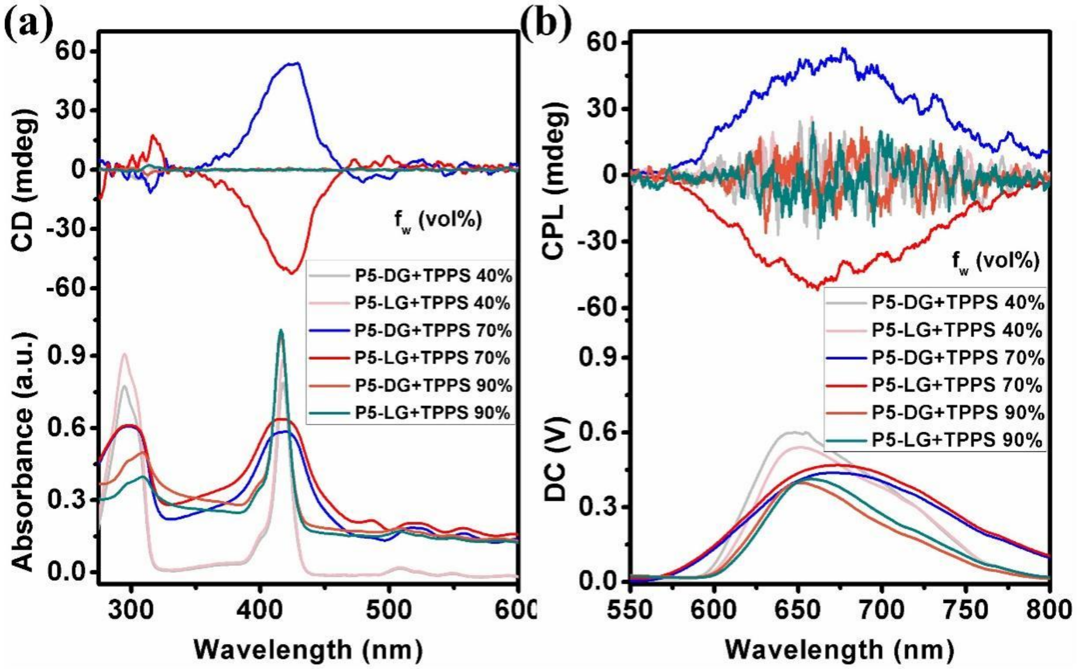

Figure S29 (a) CD spectra (top) and UV–vis absorption (Abs) (bottom) and (b) CPL (Ex = 420 nm) spectra (top) and DC value (bottom) of various topological structures of **P5-DG/LG** after adsorption of TPPS, and the cuvette path length is 0.1 mm.

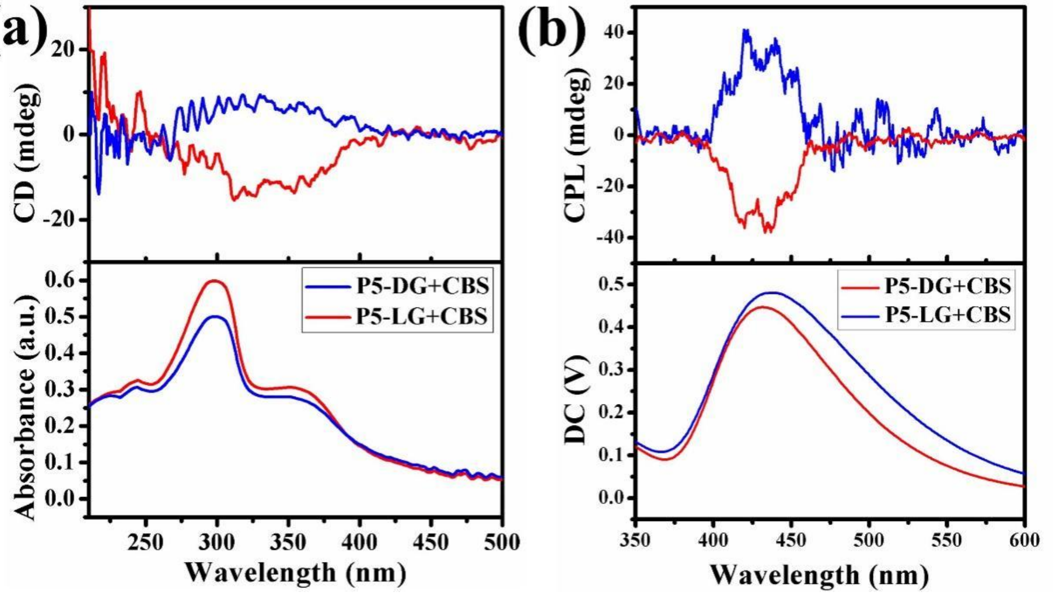

Figure S30 (a) CD spectra (top) and UV–vis absorption (Abs) (bottom) of **P5-DG/LG + CBS** in THF/H_2O (v/v=3/7), and the cuvette path length is 0.1 mm, (b) CPL (Ex = 300 nm) spectra (top) and DC value (bottom) of **P5-DG/LG + CBS** corresponding to CPL spectra.

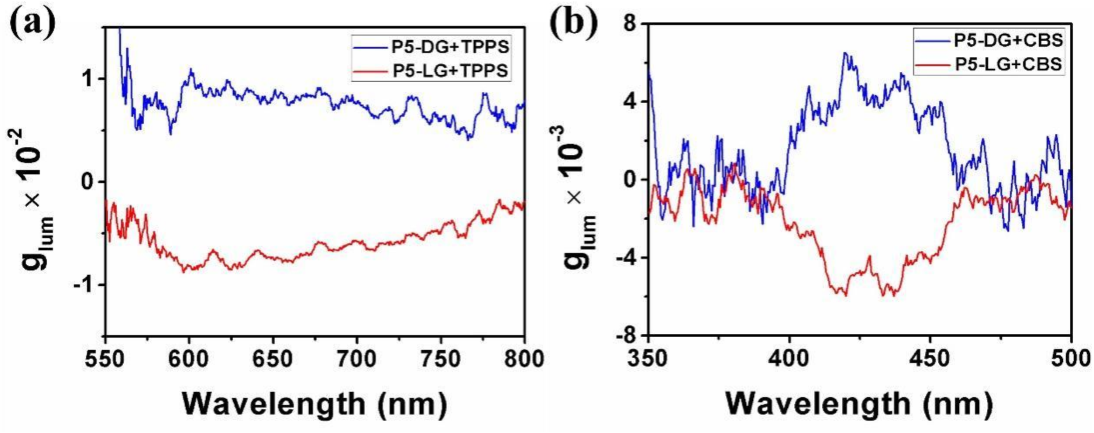

Figure S31 (a) The g_{lum} value spectra of CPL corresponding to Fig. 5e, (b) The g_{lum} value spectra of CPL corresponding to Fig. S30b.

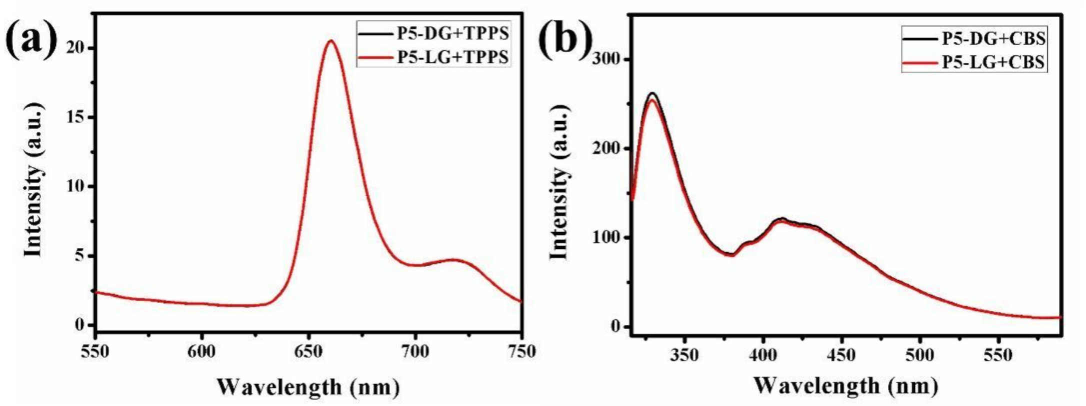

Figure S32 Emission spectra of (a) **P5-DG/LG + TPPS** (excited at 420 nm, Ex bandwidth: 5 nm; Em bandwidth: 5 nm) and (b) **P5-DG/LG + CBS** (excited at 300 nm, Ex bandwidth: 5 nm; Em bandwidth: 5 nm) in THF/H_2O (v/v=3/7).

REFERENCES

[S1] Li Y, Wang T, Liu M. *Soft Matter.* 2007, **3**, 1312-7.

[S2] P. Duan, X. Zhu, M. Liu, *Chem Commun*. 2011, **47**, 5569-71.

[S3] Frisch M J, Trucks G W, Schlegel H *et al.* Gaussian 16, Revision A.03, Gaussian, Inc., Wallingford CT, 2016.

[S4] Bannwarth C, Caldeweyher E, Ehlert S *et al*. Extended tight-binding quantum chemistry methods. *Wiley Interdiscip. Rev.: Comput. Mol. Sci.* 2021; **11**: e1493.

[S5] VandeVondele J, Krack M, Mohamed F *et al*. Quickstep: fast and accurate density functional calculations using a mixed Gaussian and plane waves approach. *Comput. Phys. Commun.* 2005; **167**: 103–28.

[S6] Kuehne T D, Iannuzzi M, Del Ben M *et al*. CP2K: an electronic structure and molecular dynamics software package—quickstep: efficient and accurate electronic structure calculations. *J. Chem. Phys.* 2020; **152**: 194103.

[S7] Lu T, Chen Q X. Interaction region indicator: a simple real space function clearly revealing both chemical bonds and weak interactions. *Chemistry-Methods.* 2021; **1**: 231–9.

[S8] Lu T, Chen F. Multiwfn: A multifunctional wavefunction analyzer. *J. Comput.*

Chem. 2012; **33**: 580-92.

[S9] Humphrey W, Dalke A, Schulten K. VMD: visual molecular dynamics. *J. Mol. Graphics Modell.* 1996; **14**: 33-8.

Author Contributions

Minghua Liu supervised the project. Jie Lu designed the experiments and synthesized the compounds. Jie Lu carried out all the charaterizations and colleted the data. Yuan Wang carried out DFT computation. Jingjun Jin carried out TEM characterization. Jie Lu and Minghua Liu analyzed the data. Jie Lu wrote the manuscript. Jie Lu, Yuan Wang and Minghua Liu revised the manuscript. All the authors participated in the discussion, interpretation of the data.
